# Physiological and transcriptome analyses reveal the photosynthetic response to drought stress in drought-sensitive (Fengjiao) and drought-tolerant (Hanjiao) *Zanthoxylum bungeanum* cultivars

**DOI:** 10.3389/fpls.2022.968714

**Published:** 2022-09-06

**Authors:** Haichao Hu, Beibei He, Lei Ma, Xin Chen, Peilin Han, Yingli Luo, Yonghong Liu, Xitong Fei, Anzhi Wei

**Affiliations:** ^1^College of Forestry, Northwest Agriculture and Forestry University, Xianyang, Shaanxi, China; ^2^Research Centre for Engineering and Technology of Zanthoxylum State Forestry Administration, Xianyang, Shaanxi, China; ^3^College of Horticulture, Northwest Agriculture and Forestry University, Xianyang, Shaanxi, China

**Keywords:** *Zanthoxylum bungeanum*, photosynthetic rate, drought stress, transcriptome, WGCNA, transcription factor

## Abstract

As an important economical plant, *Zanthoxylum bungeanum* is widely cultivated in arid and semi-arid areas. The studies associated with photosynthesis under drought stress were widely carried out, but not yet in *Z. bungeanum*. Here, the photosynthesis of two *Z. bungeanum* cultivars (FJ, *Z. bungeanum* cv. “Fengjiao”; HJ, *Z. bungeanum* cv. “Hanjiao”) was analyzed under drought stress using physiological indicators and transcriptome data. Drought decreased stomatal aperture and stomatal conductance (Gsw), reduced transpiration rate (E) and sub-stomatal CO_2_ concentration (Ci), and lowered chlorophyll and carotenoid content, which reduced the net photosynthetic rate (Pn) of *Z. bungeanum*. The higher photosynthetic rate in HJ stemmed from its higher chlorophyll content, larger stomatal aperture and Gsw, and higher Ci. Weighted gene co-expression network analysis (WGCNA) identified several ABA signal transduction genes (*PYL4*, *PYL9*, and *PYR1*), LCH-encoding genes (*LHCB4.3*), and chlorophyll metabolism genes (*CRD1*, *PORA*, and *CHLH*). Additionally, seven transcription factor genes were identified as important factors regulating photosynthesis under drought conditions. In general, a photosynthetic response model under drought stress was built firstly in *Z. bungeanum*, and the key genes involved in photosynthesis under drought stress were identified. Therefore, the results in our research provide important information for photosynthesis under drought and provided key clues for future molecular breeding in *Z. bungeanum*.

## Introduction

Drought stress is one of the most important environment factors, which severe affected the growth and development of plants (Fathi and Barari, 2016). With the global warming in recent years, water shortage has become a large challenge for sustainable agriculture and attracted wide attentions from researchers (Dai, 2013). Plants have evolved various acclimation responses to harsh environments (Zhang et al., 2022). In response to drought stress, acclimations of plant mainly include lateral root growth, stomatal closure and leaf rolling (Basu et al., 2016). However, the acclimation generally comes at the cost of plant growth and development, and lead to weakness of many metabolic processes.

Photosynthesis is the most important metabolic process for carbon assimilation in plants and the fixed biochemical energy in photosynthesis was used to support nearly all life on Earth (Evans, 2013). Photosynthesis is divided into two stages: light reactions taking place in thylakoids and dark reactions occurring in chloroplast stroma (Kaiser et al., 2015). There are four photosynthetic protein complexes located in thylakoid membranes: photosystem I (PSI), photosystem II (PSII), the cytochrome b6f complex (Cytb6f), and adenosine triphosphate (ATP) synthase (van Bezouwen et al., 2017). PSI and PSII, which comprise core complexes and peripheral antenna systems, function in light capture and subsequent photochemical reactions (Huang et al., 2021). Light-harvesting chlorophyll-binding I (LHCI) and light-harvesting chlorophyll-binding II (LHCII) serve as light-absorbing antenna systems in PSI and PSII, respectively. The pigments in LHCI and LHCII are mainly chlorophyll *a* and chlorophyll *b*, respectively (Rochaix, 2013). The light energy is absorbed by LHCs and subsequently transferred to the photosynthesis reaction center (Huang et al., 2021). Photosynthesis is highly sensitive to water deficit, which is generally weakened under drought stress (Ma et al., 2016; Zhang et al., 2021). As known, stomata are highly sensitive to drought stress (Zhang et al., 2021). Given that stomata are channels for gas exchange in photosynthesis, closure or partial closure of stomata by drought stress largely reduced intercellular carbon dioxide (CO_2_) concentration and sequenced inhibited photosynthesis. On the other hand, drought stress stimulated the accumulation of reactive oxygen species (ROS; Zhang et al., 2022). The excessive ROS destroyed pigments in photosystem and induced membrane peroxide, further inhibiting the photosystem II (PSII) activity (Gururani et al., 2015). Generally, drought-tolerant plants have higher water usage efficiency and a superior ROS-scavenging ability (Ma et al., 2016). Therefore, drought-tolerant plants typically have higher photosynthetic efficiency under drought conditions. Given that maintaining normal rates of photosynthesis under drought stress is important for ensuring high crop quality and yield (Lima-Melo et al., 2021), a large scale of researches have been carried out to explore the photosynthetic metabolism under drought stress. For instance, Ma et al. reported that the chlorophyll content in alfalfa declines significantly under drought stress (Ma et al., 2021). Zhang et al. explored the effects of drought on photosynthesis by measuring photosynthetic parameters and conducting transcriptome analysis in *Atractylodes lancea* (Zhang et al., 2021). Daszkowska-Golec et al. analyzed the physiological and genetic basis of photosynthesis in barley under drought stress (Daszkowska-Golec et al., 2019). Hong et al. found that chloroplast protein, PsbP domain protein 5 (PPD5), plays an important role in *Arabidopsis* under drought stress (Hong et al., 2020). D’Alessandro et al. revealed an important role of β-carotene oxidation in PSII in mediating stress tolerance, including drought tolerance (D'Alessandro and Havaux, 2019).

*Zanthoxylum* belongs to the family Rutaceae and is widely distributed in Asia countries, including China, Korea, Japan and India. Over 250 species to date have been identified in *Zanthoxylum* genus across the world, of which the most well-known species are *Zanthoxylum bungeanum* (Red Huajiao) and *Zanthoxylum armatum* (Green Huajiao; Wang et al., 2022). The pericarp of *Z. bungeanum* is an essential culinary spice and condiment in Chinese cuisine, including the well-known Sichuan hot pot (Okagu et al., 2021a). Additionally, *Z. bungeanum* pericarp is a kind of traditional Chinese medicine, with numerous biological functions in anti-inflammatory, analgesic, antimicrobial, and antiviral activities (Okagu et al., 2021b; Wang et al., 2022). *Zanthoxylum bungeanum* plant has evolved a large scale of prickles, which are distributed in the stems, branches, as well as leaves (Zhang et al., 2017). Like other prickle plants, *Z. bungeanum* plant exhibits a strong acclimation response to drought environment. Due to their economic value and drought tolerance, *Z. bungeanum* plants are widely grown in arid and semi-arid areas. In recent years, increasing researches have focused on *Z. bungeanum*, including the chemical component of *Z. bungeanum* pericarp (Fei et al., 2021), the disease control of *Z. bungeanum* plant (Li et al., 2021b) and *Z. bungeanum* genome sequencing for evolutionary relationship (Feng et al., 2020). In addition, the response mechanism of prickly ash plants underlying chilling injury has been preliminarily explored (Tian et al., 2021). However, response mechanism of photosynthesis under drought stress of *Z. bungeanum* remains unexplored and is urgent to reveal.

Here, we investigated the influence of drought stress on photosynthetic parameters, stomatal status and chlorophyll content of two different *Z. bungeanum* cultivars with diverse drought tolerance under progressive drought stress. Meanwhile, we explored the underlying mechanisms of photosynthesis variation through transcriptome analysis. Furthermore, we screened many structural genes and transcription factors (TFs) closely associated with the photosynthesis of *Z. bungeanum* plants through weighted gene co-expression network analysis (WGCNA). Therefore, our study provides important information for molecular breeding of *Z. bungeanum* plants.

## Materials and methods

### Plant material and sample

The mature seeds of a drought-sensitive cultivar (FJ, *Z. bungeanum* cv. “Fengjiao”) and a drought-tolerant cultivar (HJ, *Z. bungeanum* cv. “Hanjiao”) were harvested in the Fengxian Prickly Ash Experimental Station of Northwest A&F University in Shannxi Province, China (33°59′6.55′′N, 106°39′29.38′′E). The *Z. bungeanum* seeds were cleaned, air-dried, and sown in a cultivar soil mixture consisting of perlite, vermiculite, and chernozem in a research greenhouse of Northwest A&F University in Yangling, Shannxi Province, China. One week after germination, healthy seedlings were transplanted to cultivar pots for cultivation at 25°C ± 2°C and soil moisture of 85% ± 1%. After 3 months, 54 *Z. bungeanum* healthy seedlings of the same size of each cultivar were subjected to drought treatment (i.e., these plants were not provided water) for 15 days. On 0, 3, 6, 9, 12, and 15 days after the start of the drought treatment, leaf samples of *Z. bungeanum* seedlings were obtained, dipped into liquid nitrogen, and stored in a −80°C freezer. There were three biological replicates for each sample and three seedlings for each biological replicate.

### Photosynthetic indicators

The net photosynthetic rate (Pn), transpiration rate (E), stomatal conductance (Gsw), and intercellular CO_2_ concentration (Ci) of *Z. bungeanum* seedlings were measured using an LI-6800 photosynthesis measurement system (LI-COR, Lincoln, NE, United States) between 8:30 and 11:30 AM at 0, 3, 6, 9, 12, and 15 days of drought treatment. All measurements were performed on the third fully expanded mature leaves from the top of *Z. bungeanum* seedlings, and a total of nine seedlings were analyzed per cultivar.

### Stomatal morphology

The third fully expanded leaves from the top of the seedlings were used for observations of stomatal morphology. Transparent acrylic nail polish was applied to the lower epidermis of *Z. bungeanum* leaves following the methods described in a previous study (Sun et al., 2020). When the nail polish was dried, it was gently peeled off with tweezers, placed on a glass slide, and covered with a cover slip. A fluorescence microscope (BX63, Olympus, Japan) was then used to take photographs of the slide at a 10 × 40 magnification. There were three epidermis samples per plant, and there were two fields of view of each epidermis sample. Stomatal density was calculated as the number of pores per unit area. Five stomata were randomly selected from each field of view for length and width measurements in ImageJ version 1.48 (National Institutes of Health, Bethesda, MD, United States). The stomatal aperture was indicated by the width of the stomata (Supplementary Figure 1).

### Measurements of photosynthetic pigments from fresh leaves

Extractions and measurements of chlorophyll and carotenoids were performed following a previously described method (Xue et al., 2020) with some modifications. Briefly, 0.05 g of fresh leaves were ground into powder with liquid nitrogen and fully mixed with 8 ml of 80% acetone. After samples were on ice for 20 min, the homogenate was filtered through filter paper. The absorbance of the supernatants was measured at 652.4, 665.2, and 470 nm using a microplate reader (Infinite M200pro, Tecan, Switzerland). The content of photosynthetic pigments was calculated using the following equations:

Chlorophyll *a* = 16.72 A665.2 – 9.16 A652.4

Chlorophyll *b* = 34.09 A652.4 – 15.28 A665.2

Total chlorophyll = chlorophyll *a* + chlorophyll *b*

Carotenoid = (1,000 A470 – 1.63 chlorophyll *a*  − 104.96 chlorophyll *b*)/221.

### RNA extraction and sequencing

The leaf samples of two *Z. bungeanum* cultivars obtained on 0 (D1), 6 (D2), 9 (D3), and 15 days (D4) were used for RNA extraction and sequencing. We extracted the total RNA from the *Z. bungeanum* leaves using the Tiangen RNA Pure kit for plants (Tiangen, Beijing, China). The purity and integrity of extracted RNA were determined on an Agilent 2,100 Bioanalyzer (Agilent Technologies, Inc., Santa Clara, CA, United States). RNA with an OD260/280 value between 1.8 and 2.2 and OD260/230 value over 2.0 was used for the construction of cDNA libraries with the NEBNext Ultra RNA Library Prep Kit for Illumina (New England Biolabs, Ipswich, United States). The resulting libraries were sequenced using the Illumina HiSeq 2,500 platform (Illumina, Inc., San Diego, United States).

RNA sequencing reads were aligned to the reference *Z. bungeanum* genome using HISAT2 (Kim et al., 2015). FPKM (Fragments Per Kilobase of transcript per Million fragments mapped) was calculated using StringTie (Pertea et al., 2015). Genes with fold change (FC) ≥ 2 and false discovery rate (FDR) < 0.01 were defined as differentially expressed genes (DEGs). The functions of genes were determined using the Gene Ontology (GO), Kyoto Encyclopedia of Genes and Genomes (KEGG), Clusters of Orthologous Groups of proteins (KOG/COG), Protein family (Pfam), Swiss-Prot (a manually annotated and reviewed protein sequence database), NCBI non-redundant protein sequence (Nr), and NCBI non-redundant nucleotide sequence (Nt) database.

### Weighted gene co-expression network analysis

Weighted gene co-expression network analysis (WGCNA) was carried out on the DEGs using R (version: 1.70–3) with default parameters (Langfelder and Horvath, 2008). The photosynthetic indicators were input as the trait file. The top 150 core genes in the green, purple and cyan modules were used to construct the co-expression network and visualized using Cytoscape 3.9.1 (Java 11.0.6).

### Phylogenetic analysis and *cis*-element analysis of seven TF genes

The phylogenetic tree was built based on the amino acid sequences of seven TFs using the neighbor-joining (NJ) method in MEGA 7.0. The upstream 2,000-bp (bp) sequences of the start codon were extracted from the 13 TF genes using TBtools, which were then submitted to the PlantCARE database (http://bioinformatics.psb.ugent.be/webtools/plantcare/html/, accessed on 25 May 2022). The file generated from PlantCARE was used to characterize the *cis*-element distribution using TBtools (Chen et al., 2020).

### Quantitative real-time PCR analysis

A Tiangen RNA Pure kit for plants (Tiangen, Beijing, China) was used to extract the total RNA in *Z. bungeanum* leaves. A NanoDrop 2000 spectrophotometer (Thermo Scientific, Wilmington, DE, United States) was used to analyze the quality of the total RNA; RNA with an OD260/280 value between 1.8 and 2.2 and OD260/230 value over 2.0 was reverse-transcribed into first-strand cDNA using the PrimeScript RT Reagent Kit with gDNA Eraser (Takara Biotechnology Inc., Dalian, China). qRT-PCR was carried out in a volume of 10 μl using the SYBR^®^ Green Premix *Pro Taq* HS qPCR Kit (Accurate Biotechnology Co., Ltd., Hunan, China) on a CFX96 Real-Time System (Bio-Rad Laboratories, Inc., Hercules, United States). The quantitative primers in qRT-PCR were designed using Primer Premier 6.0 (PREMIER Biosoft, CA, United States; Supplementary Table 1). *ZbUBA* and *ZbUBQ* were used as reference genes (Fei et al., 2018), and relative expression levels were calculated using the 2^−ΔΔCT^ method (Schmittgen and Livak, 2008).

### Statistical analysis

The experimental data included three biological replicates, and significant differences were analyzed using one-way ANOVA, followed by Duncan’s multiple-range test (*p <* 0.05), in SPSS 23.0 software (SPSS Inc., Chicago, IL, United States). Line charts and column charts were made using OriginPro 2021 (Originlab, Northampton, MA, United States). The inter-group correlation analysis was conducted, and the results were visualized using OriginPro 2021 (Originlab, Northampton, MA, United States). Venn diagrams and volcano diagrams were made using the BMKCloud platform.^1^

## Results

### Morphological traits and stomatal characteristic of *Zanthoxylum bungeanum* leaves under drought stress

**Figure 1:**
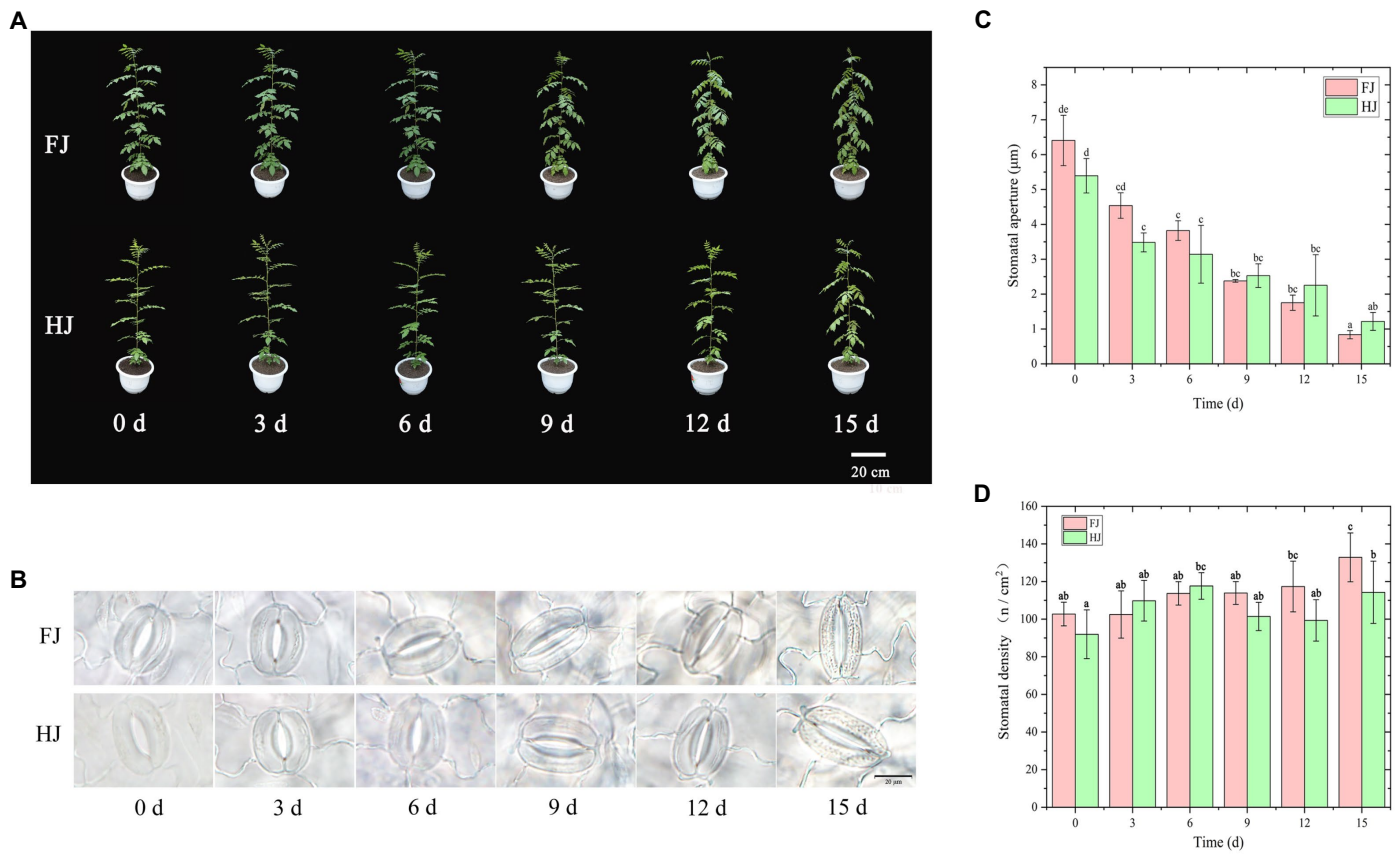
Morphological traits and stomatal characteristic of *Zanthoxylum bungeanum* leaves under drought stress. **(A)** Morphological traits of *Z. bungeanum* seedlings under progressive drought stress. **(B)** Images of the stomata and guard cells of *Z. bungeanum* leaves taken with a fluorescence microscope. **(C)** Stomatal aperture. **(D)** Stomatal density. All data are mean ± standard error of three replicates. Values with different letters indicate significant differences at *p* < 0.05 between FJ and HJ based on one-way ANOVA.

The morphological traits of two *Z. bungeanum* cultivars under progressive drought stress are displayed in Figure 1A. The *Z. bungeanum* seedlings changed slightly before 6 days in both FJ and HJ. In FJ, the drought-sensitive cultivar, the leaves began to roll in at 9 days and such case became worse over time. However, the leaves roll in slightly at 12 and at 15 days in HJ, the drought-tolerant cultivar. Notably, the changes of morphological traits were much slighter in HJ than in FJ at each timepoint.

Stomata are an important kind of channel for gas exchange and an important basis for photosynthesis. Here, morphological characteristic of the stomata of *Z. bungeanum* leaves were observed under progressive drought stress using a fluorescence microscope (Figure 1B). The result showed that the stomatal morphology was significantly changed by drought stress. The stomatal aperture decreased with drought stress in both *Z. bungeanum* cultivars, with largest value in 0d (FJ, 6.41 μm; HJ, 5.39 μm) and smallest at 15 days (FJ, 0.72 μm; HJ, 1.22 μm; Figure 1C). The stomatal aperture was higher before 6 days and lower after 9 days in FJ than in HJ. The stomatal density increased steadily and was largest at 15 days (136.17/cm^2^) in FJ (Figure 1D). However, in HJ, stomatal density reached a peak value at 6 days (117.64/cm^2^) and then decreased until 12 days. Overall, drought stress decreased the stomatal aperture and increased the stomatal intensity in *Z. bungeanum* leaves.

### Photosynthetic parameters of *Zanthoxylum bungeanum* leaves under drought stress

**Figure 2:**
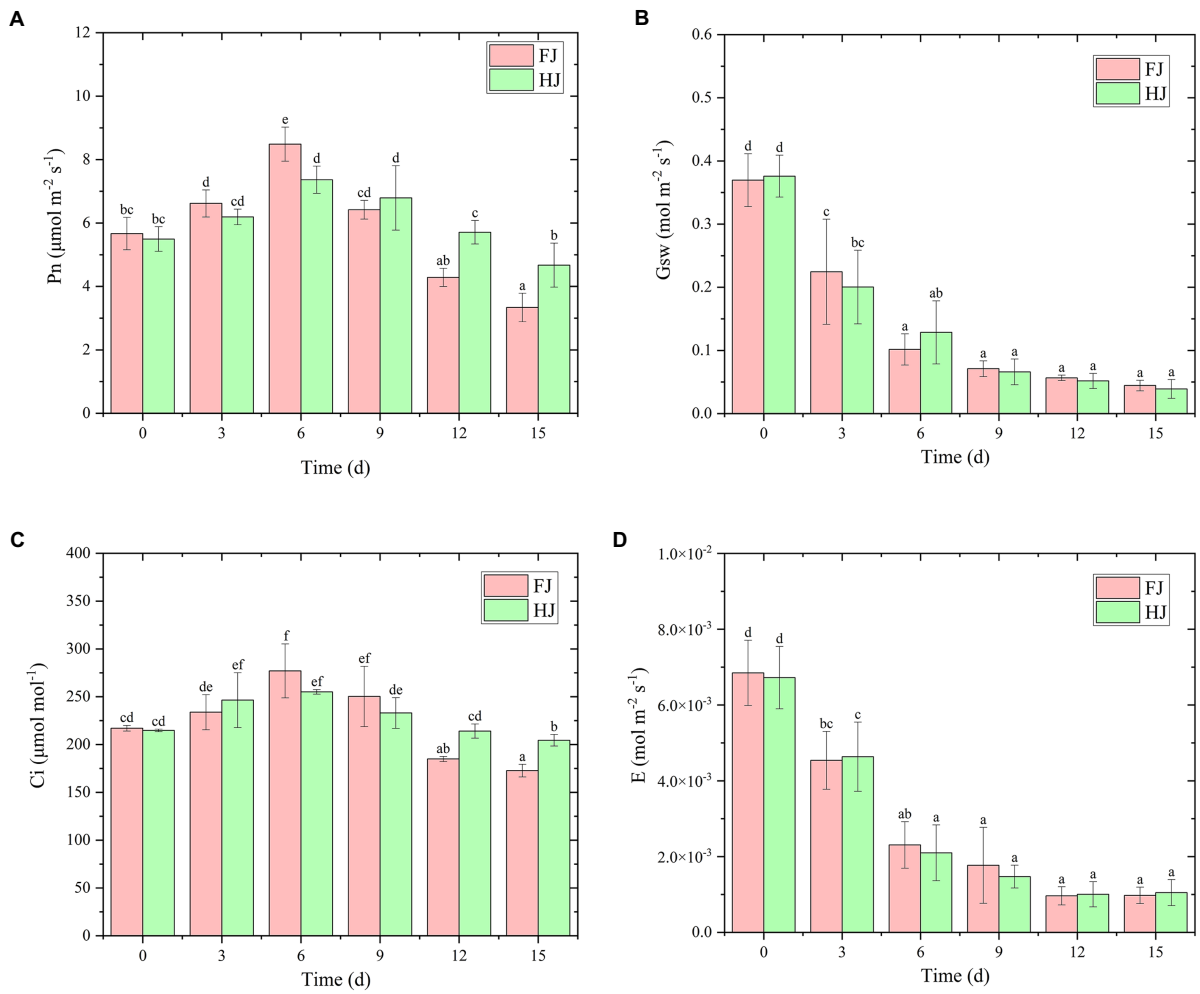
Photosynthetic parameters of two *Zanthoxylum bungeanum* cultivars under drought stress. **(A)** Net photosynthetic rate (Pn). **(B)** Stomatal conductance (Gsw). **(C)** Sub-stomatal CO_2_ concentration (Ci). **(D)** Transpiration rate (E). All data are mean ± standard error of three replicates. Values with different letters indicate significant differences at *p* < 0.05 between FJ and HJ based on one-way ANOVA.

To explore the influence of drought stress on photosynthesis in *Z. bungeanum* plants, photosynthetic parameters, including the net photosynthetic rate (Pn), stomatal conductance (Gsw), sub-stomatal CO_2_ concentration (Ci) and transpiration rate (E), were measured using a Li 6,800 photosynthesis measurement system (Figure 2). The Pn reached a peak value in both cultivars at 6 days (FJ, 8.48 μmol m^−2^ s^−1^; HJ, 7.36 μmol m^−2^ s^−1^) and then decreased steadily (Figure 2A). The stimulation of photosynthesis may be used to mitigate light damage caused by drought in the early stage. At 6 days, Pn was significantly higher in FJ than in HJ (*p* < 0.05), but after that it was lower in FJ than in HJ, extremely for 12 and 15days (*p* < 0.05), indicating that drought made more pronounced influence on FJ than on HJ in late stage of drought treatment. Similarly, Ci increased dramatically when suffering the drought stress and peaked at 6 days in FJ (277.01 μmol mol^−1^) and in HJ (255.09 μmol mol^−1^; Figure 2C). At days 12 and 15, the Ci was significantly lower in FJ than in HJ (*p* < 0.05). The Gsw and E decreased significantly at the start of the drought stress and varied slightly after 6 days (Figures 2B,D). Taken together, drought stress negatively affected the photosynthetic efficiency in both cultivars, especially after 6 days of drought treatment.

### The contents of chlorophyll and carotenoid in *Zanthoxylum bungeanum* leaves under drought stress

**Figure 3:**
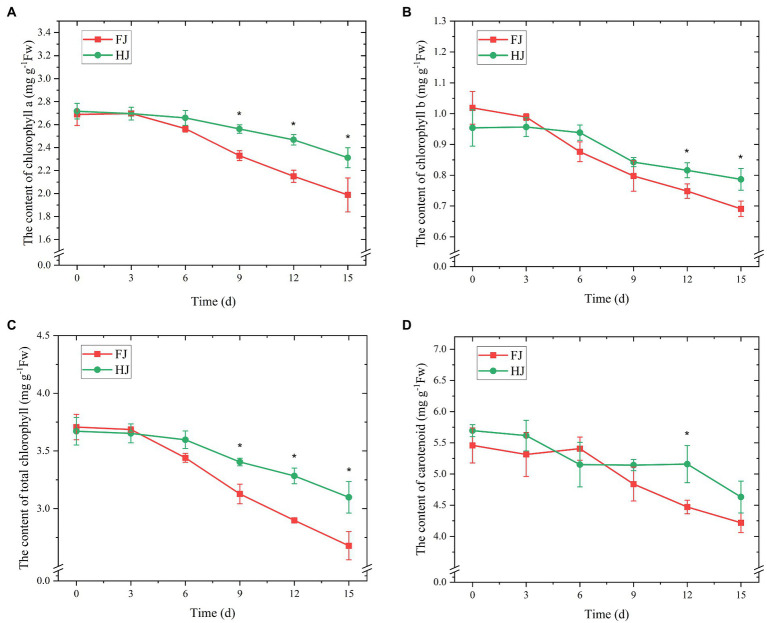
The content of chlorophyll *a* **(A)**, chlorophyll *b* **(B)**, total chlorophyll **(C)**, and carotenoids **(D)** of *Zanthoxylum bungeanum* leaves under drought stress. All data are mean ± standard error of three replicates. Values with asterisks indicate significant differences at *p* < 0.05 between FJ and HJ based on one-way ANOVA.

Given the key role of chlorophyll and carotenoid in plant photosynthesis system, we monitored their content variations under progressive drought conditions. The content of chlorophyll *a*, chlorophyll *b*, total chlorophyll and carotenoid decreased under drought stress in both cultivars (Figure 3). The content of chlorophyll *a* varied slightly before 6 days and decreased dramatically after 6 days in both FJ and HJ (Figure 3A). The content of chlorophyll *a* was higher in HJ than in FJ after 9 days (*p* < 0.05). The content of chlorophyll *b* decreased dramatically after 3 days in FJ and after 6 days in HJ (Figure 3B). The content of chlorophyll *b* was significantly lower in FJ than in HJ on 12 and 15 days (*p* < 0.05). In terms of the content of total chlorophyll, it was higher in HJ than in FJ on days 9, 12, and 15 (*p* < 0.05; Figure 3C). Carotenoid content varied slightly in FJ before 6 days and decreased significantly after that (Figure 3D). Compared to that in FJ, the carotenoid content in HJ was significantly higher on 12 days (*p* < 0.05). In general, drought stress decreased the contents of chlorophyll *a*, chlorophyll *b*, total chlorophyll and carotenoid in *Z. bungeanum* plants and these contents in HJ were relatively higher than in FJ in the late drought stage.

### Correlation analysis of photosynthesis indicators

**Figure 4:**
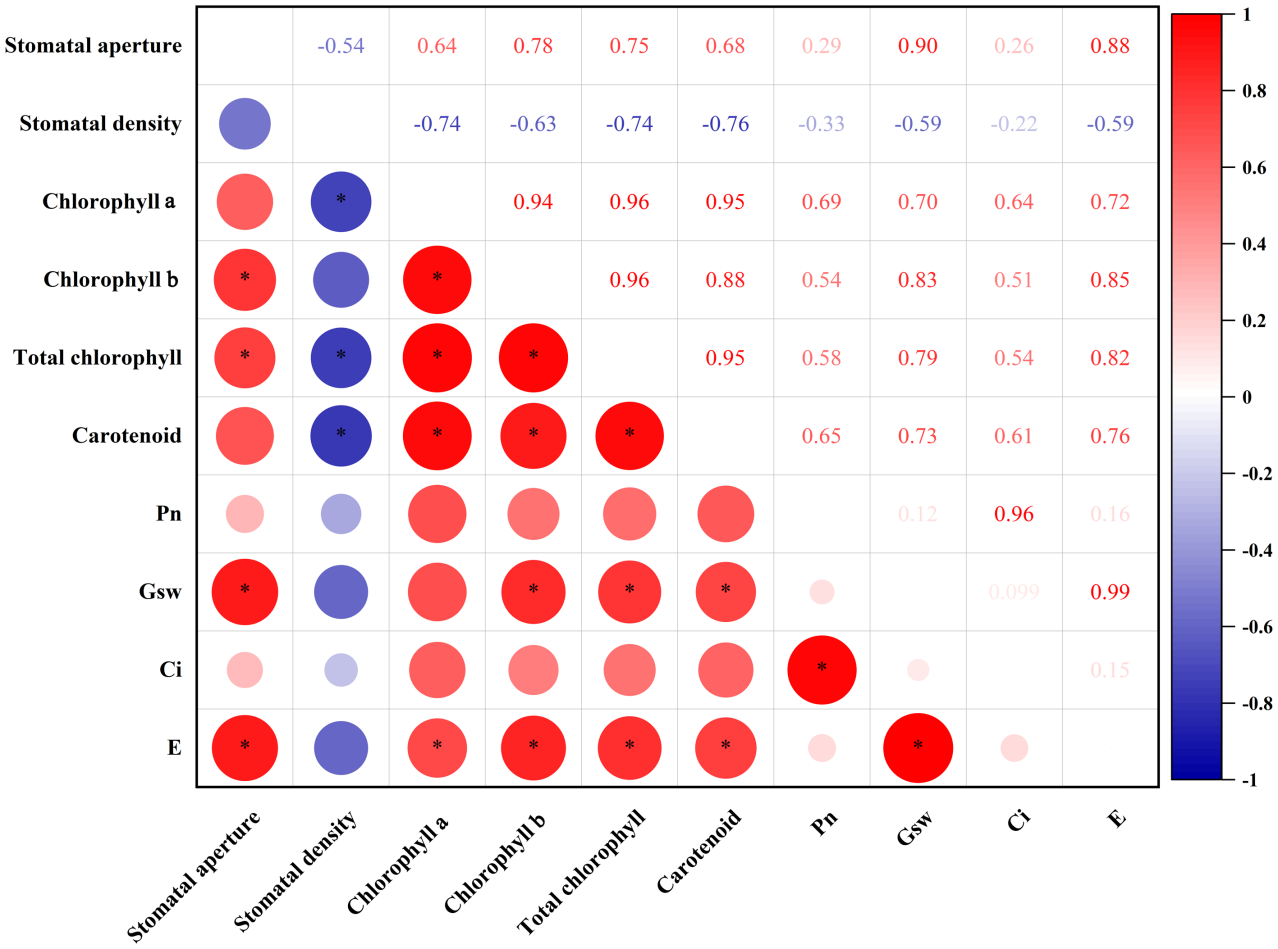
Intragroup correlation analysis of photosynthesis-related indicators. The size of the circles in the lower triangles indicates the strength of the correlation. Red indicates positive correlations; blue indicates negative correlations. Significant correlations (*p* < 0.05) are indicated by asterisks. The numbers in the upper triangles indicate the correlation coefficients between two indicators. Pn, net photosynthetic rate; Gsw, stomatal conductance; Ci, sub-stomatal CO_2_ concentration; and E, transpiration rate.

To clear the relationship among these photosynthesis indicators, intragroup correlation analysis was performed (Figure 4). Stomatal aperture was positively correlated with chlorophyll *b* (*R* = 0.78, *p* < 0.05), total chlorophyll (*R* = 0.75, *p* < 0.05), Gsw (*R* = 0.90, *p* < 0.05), and E (*R* = 0.88, *p* < 0.05). Stomatal density was negatively correlated with chlorophyll *a* (*R* = −0.74, *p* < 0.05), total chlorophyll (*R* = −0.74, *p* < 0.05) and carotenoid (*R* = −0.76, *p* < 0.05). In terms of chlorophyll *a*, chlorophyll *b*, total chlorophyll and carotenoid, they were positively correlated with each other, indicating they functioned synergistically in photosystem. Besides, Pn was positively related to Ci (*R* = 0.96, *p* < 0.05), which reflects the fact that intercellular CO_2_ is the raw material of photosynthesis. Gsw was highly positively related to E (*R* = 0.99, *p* < 0.05), in line with that stomata status is extremely important for plant transpiration.

### Differentially expressed genes in FJ and HJ under drought stress

To explore the molecular mechanisms underlying the photosynthesis changes under drought, transcriptome sequencing was performed at four drought stages: D1 (0 days), D2 (6 days), D3 (9 days), and D4 (15 days), respectively. A total of 169.59 Gb clean data were obtained, with more than 5.82 Gb clean data in each sample.

**Figure 5:**
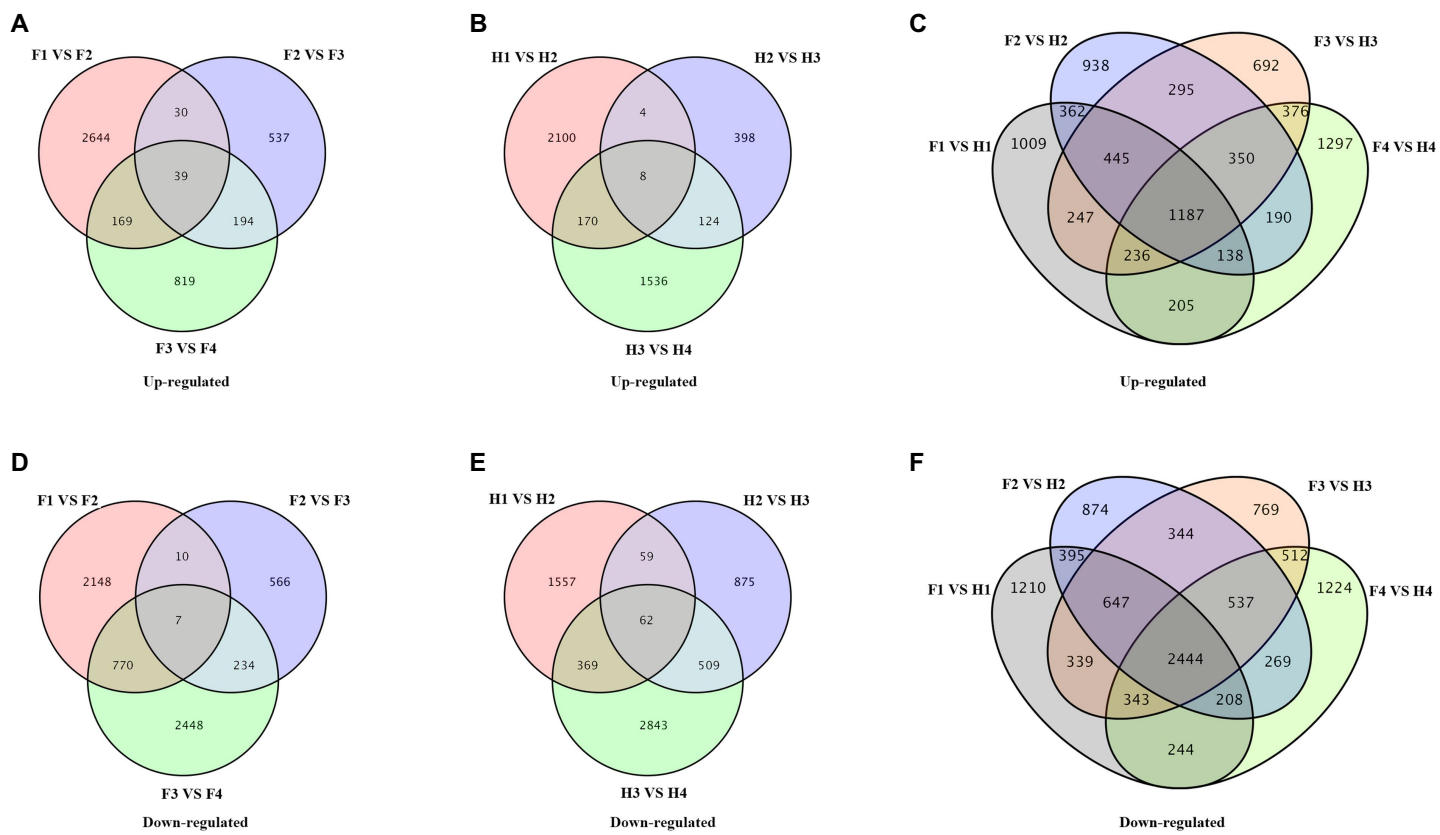
Venn diagrams of differentially expressed genes (DEGs) in different comparisons. **(A–C)** Venn diagrams of upregulated genes. **(D–F)** Venn diagrams of downregulated genes. F1, F2, F3, and F4 indicate FJ samples in 0, 6, 9, and 15 days under drought stress; H1, H2, H3, and H4 indicate HJ samples in 0, 6, 9, and 15 days under drought stress.

The differentially expressed genes (DEGs) in different comparisons were shown in Venn diagrams (Figure 5). In FJ, there were 2,882 upregulated genes and 2,935 downregulated genes in F1 vs. F2, 800 upregulated genes and 817 downregulated genes in F2 vs. F3, and 1,221 upregulated genes and 3,459 downregulated genes in F3 vs. F4 (Figures 5A,D). In HJ, there were 2,282 upregulated genes and 2,047 downregulated genes in H1 vs. H2, 534 upregulated genes and 1,505 downregulated genes in H2 vs. H3, and 1,838 upregulated genes and 3,783 downregulated genes in H3 vs. H4 (Figures 5B,E). There was a greater number of upregulated genes in FJ than in HJ in D2 and D3, indicating more stable homeostasis in HJ in the early stage. Besides, the DEGs between FJ and HJ in each time were also analyzed (Figures 5C,F). There were 2,282 upregulated genes and 2,047 downregulated genes in F1 vs. H1, 534 upregulated genes and 1,505 downregulated genes in F2 vs. H2, 1,838 upregulated genes and 3,783 downregulated genes in F3 vs. H3, and 534 upregulated genes and 1,505 downregulated genes in F4 vs. H4.The overlap of DEGs across all stages can provide insight into the response mechanism of plants to drought stress. The overlap analysis revealed that there were 39 upregulated genes and seven downregulated genes over the four stages in FJ and eight upregulated genes and 62 downregulated genes over the four stages in HJ.

**Figure 6:**
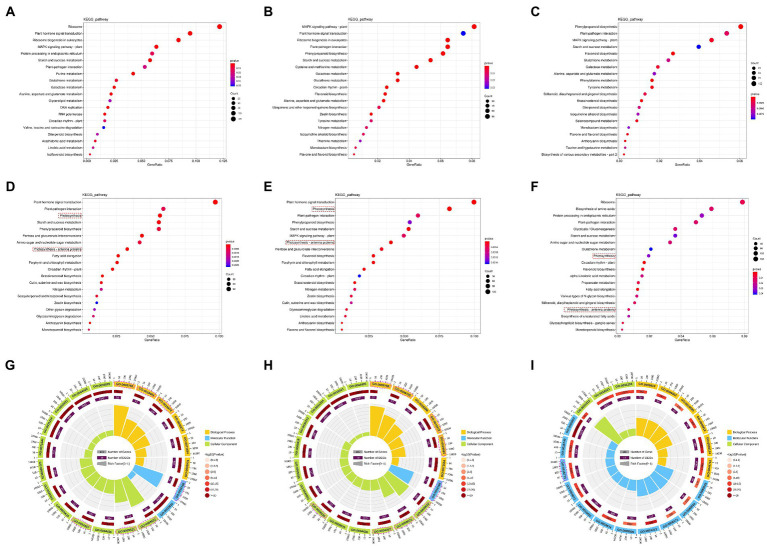
KEGG and GO enrichment analysis of DEGs in FJ and HJ. **(A)** Bubble diagram of the top 20 KEGG pathways of the upregulated genes in FJ. Bubble size indicates the number of DEGs enriched in KEGG pathways; bubble color indicates *p*-values. **(B)** Bubble diagram of the top 20 KEGG pathways of upregulated genes in FJ. **(C)** Bubble diagram of the top 20 KEGG pathways of upregulated genes in FJ vs. HJ. **(D)** Bubble diagram of the top 20 KEGG pathways of downregulated genes in FJ. **(E)** Bubble diagram of the top 20 KEGG pathways of downregulated genes in HJ. **(F)** Bubble diagram of the top 20 KEGG pathways of downregulated genes in FJ vs. HJ. **(G)** GO enrichment circle diagram of downregulated genes in FJ. The outer ring indicates the top 20 GO terms; the middle ring indicates the numbers of all genes in the GO terms and *p*-values for gene enrichment for the specified GO terms; and the inner ring indicates the numbers of DEGs. The ladder column in the center indicates the Rich factor of DEGs for each GO term. **(H)** GO enrichment circle diagram of downregulated genes in HJ. **(I)** GO enrichment circle diagram of downregulated genes in FJ vs. HJ. The pink dotted box and circle indicate the photosynthetic KEGG pathway and GO terms, respectively.

To further explore the function of the DEGs, GO and KEGG enrichment analysis were performed (Figure 6). The upregulated genes were mainly enriched on hormone signaling transportation in both FJ and HJ, such as plant hormone signal transduction (ko04075) and MAPK signaling pathway—plant (ko04016), and metabolism of metabolites, including alanine, aspartate and glutamate metabolism (ko00250), and galactose metabolism (ko00052; Figures 6A,B). Interestingly, two photosynthesis related pathways, named photosynthesis (ko00195) and photosynthesis—antenna proteins (ko00196), were significantly enriched by downregulated genes in both FJ and HJ (Figures 6D,E). Between FJ and HJ, the DEGs with upregulation in HJ were mainly enriched in flavonoid biosynthesis (ko00941), brassinosteroid biosynthesis (ko00905), phenylpropanoid biosynthesis (ko00940) and MAPK signaling pathway—plant (ko04016; Figure 6C). The DEGs with downregulation in HJ were significantly enriched in biosynthesis of amino acids (ko01230), biosynthesis of unsaturated fatty acids (ko01040), starch and sucrose metabolism (ko00500), and glutathione metabolism (ko00480; Figure 6F). The results suggested drought response metabolism may diverse between two *Z. bungeanum* cultivars. Furthermore, DEGs upregulated in F3 vs. H3 significantly enriched in anthocyanin biosynthesis (ko00942), sulfur metabolism (ko00920), phenylpropanoid biosynthesis (ko00940) and flavonoid biosynthesis (ko00941; Supplementary Figure 2A). DEGs upregulated in F4 vs. H4 significantly enriched in phenylpropanoid biosynthesis (ko00940), biosynthesis of various secondary metabolites—part 2 (ko00998), phenylalanine metabolism (ko00360), MAPK signaling pathway—plant (ko04016), brassinosteroid biosynthesis (ko00905) and tyrosine metabolism (ko00350; Supplementary Figure 2B). These results suggested that the enhanced MAPK signal transduction, the activated synthesis of brassinosteroid, and the flavonoids metabolism may be responsible for the enhanced drought tolerance of HJ in the late stage of drought.

Furthermore, GO analysis was carried out on these downregulated DEGs in FJ and HJ under drought stress and the upregulated DEGs in FJ vs. HJ (Figures 6G–I). In FJ, seven of the top 20 GO terms were associated with photosynthesis, including photosynthesis (GO:0015979), photosystem (GO:0009521), photosystem I (GO:0009522), photosynthetic membrane (GO:0034357), chlorophyll binding (GO:0016168), photosynthesis, light harvesting (GO:0009765) and photosynthesis, light reaction (GO:0019684; Figure 6G; Supplementary Table 2). There were eight GO terms included in the top 20 GO terms in HJ, consisting of the seven aforementioned GO terms in FJ and photosynthesis, light harvesting in photosystem I (GO:0009768; Figure 6H; Supplementary Table 3). The results indicated that the weakened photosynthesis rate by drought stress may be resulted from the downregulation of photosynthesis related genes. Most of the top 20 GO terms in FJ and HJ belong to cellular component, indicating drought stress greatly influenced the cellular component in *Z. bungeanum* seedlings. Differently, most of the top 20 GO terms in FJ vs. HJ belong to molecular function (Figure 6l; Supplementary Table 4).

### Weighted gene co-expression network analysis of DEGs under drought stress

**Figure 7:**
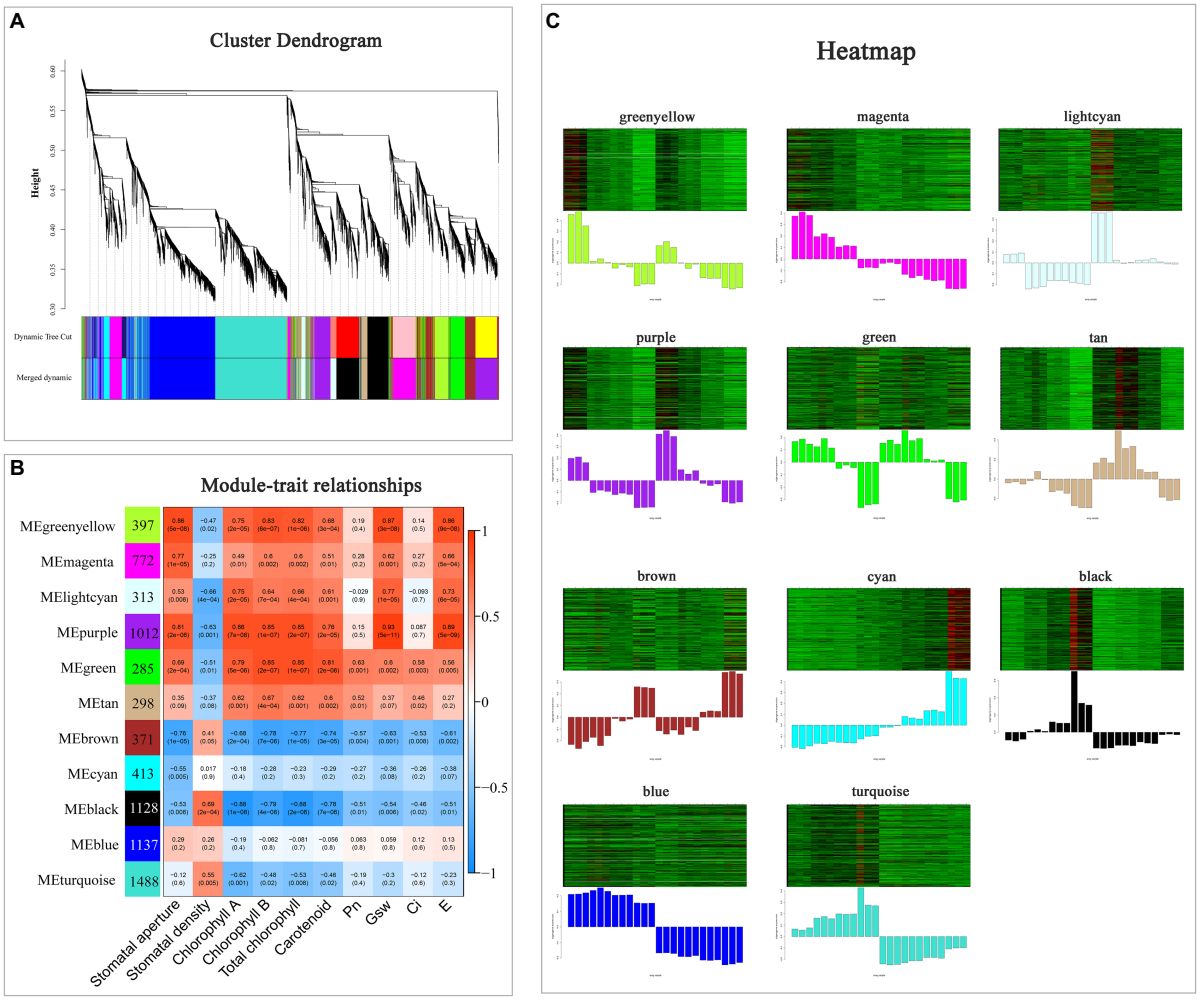
Weighted gene co-expression network analysis of DEGs under drought stress. **(A)** Cluster dendrogram of all DEGs. **(B)** Module–trait relationship heatmap of 11 modules and 10 physiological indicators. **(C)** Gene expression heatmaps of 11 modules. The column diagrams below show variation in the genes in specific modules.

WGCNA was performed based on the DEGs and physiological indicators (Figure 7). Totally, 11 modules with high gene co-expression were generated (Figure 7A). The correlation analysis among 11 modules was visualized using a heatmap (Supplementary Figure 3). There were positive correlations among the green-yellow, magenta, light-cyan, purple, green and tan modules (*R* > 0.55, *p* < 0.01).

Among these 11 modules, four contained more than 1,000 DEGs, and they were purple module (1,012 DEGs), black module (1,128 DEGs), blue module (1,137 DEGs) and turquoise module (1,488 DEGs), respectively (Figure 7B). Four modules (green-yellow, light-cyan, purple, and green) were positively correlated with most of the indicators and their expression patterns are displayed in Figure 7C (*R* > 0.55, *p* < 0.005). Among them, the purple module had a higher correlated coincidence with most indicators. Additionally, the green module was mostly positively related to Pn (*R* = 0.63, *p* = 0.001) and Ci (*R* = 0.58, *p* = 0.003). Besides, the gene expression level in cyan module increased under progressive drought and was higher in HJ than in FJ. Taken together, the green, purple and cyan modules were selected for further study.

The closure of stomata is one of the most important epigenetic changes in response to adverse environment, which is regulated by ABA. Interestingly, several genes encoding ABA signaling were included in purple module, including two *PYL4* (*EVM0086678*, *EVM0056698*), one *PYL9* (*EVM0004250*) and one *PYR1* (*EVM0057346*), and the expression level of these four genes decreased with drought treatment, which was consistent with most photosynthesis indicators (Supplementary Table 5).

### Co-expression network of DEGs in green, purple, and cyan modules

**Figure 8:**
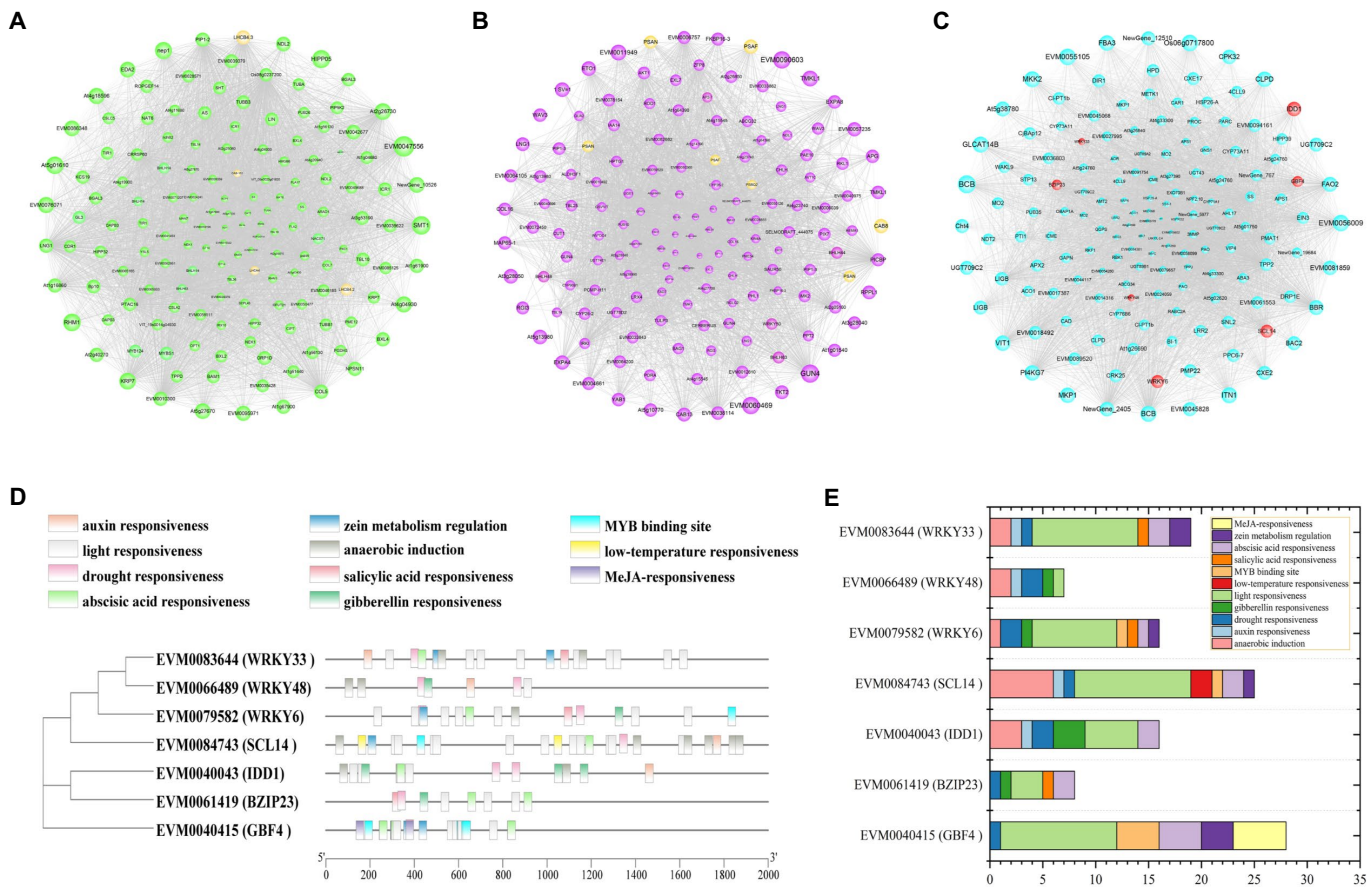
Co-expression network analysis of DEGs. **(A)** Co-expression network of 150 core DEGs in the green module. **(B)** Co-expression network of 150 core DEGs in the purple module. **(C)** Co-expression network of 150 core DEGs in the cyan module. The yellow bubbles show the genes enriched in photosynthetic related pathway. The red bubbles indicate the transcription factor genes. Bubble size indicates the connectivity degree. **(D)** *cis*-element locations in the promoters of seven TF genes identified in co-expression network in cyan module. **(E)** Statistics of *cis*-elements in each TF gene.

The co-expression networks were constructed using the top 150 core genes in green, purple, and cyan modules (Figures 8A–C). In the green module, four genes (*LHCB4.3*, *LHCA4*, *LHCB4.2*, and *CAB-151*) were enriched in photosynthesis—antenna proteins (ko00196), and one gene (*psbW*) was enriched in photosynthesis (ko00195; Supplementary Figure 4A; Supplementary Table 6). In purple module, one gene (*CAB8*) was enriched in photosynthesis—antenna proteins (ko00196), six genes (three *PSAN*s, two *PSAF*s, and one *PSBQ2*) were enriched in photosynthesis (ko00195), and six genes (three *CHLH*s, *CRD1*, *HEMA1*, and *PORA*) were enriched in porphyrin and chlorophyll metabolism (ko00860; Supplementary Figure 4B; Supplementary Table 7). The expression pattern of these 19 photosynthesis related genes was analyzed (Supplementary Figure 5). The expression level of all these genes decreased with drought and six of them showed higher expression level in HJ than in FJ under drought stress, including *PSAN* (*EVM0004914*), *LHCB4.3* (*EVM0014783*), *CHLH*s (*EVM0019653*, *EVM0057103*), *PORA* (*EVM0025664*), and *CRD1* (*EVM0071735*). Therefore, these six photosynthesis related genes may be helpful to maintain higher photosynthesis in *Z. bungeanum* seedlings.

**Figure 9:**
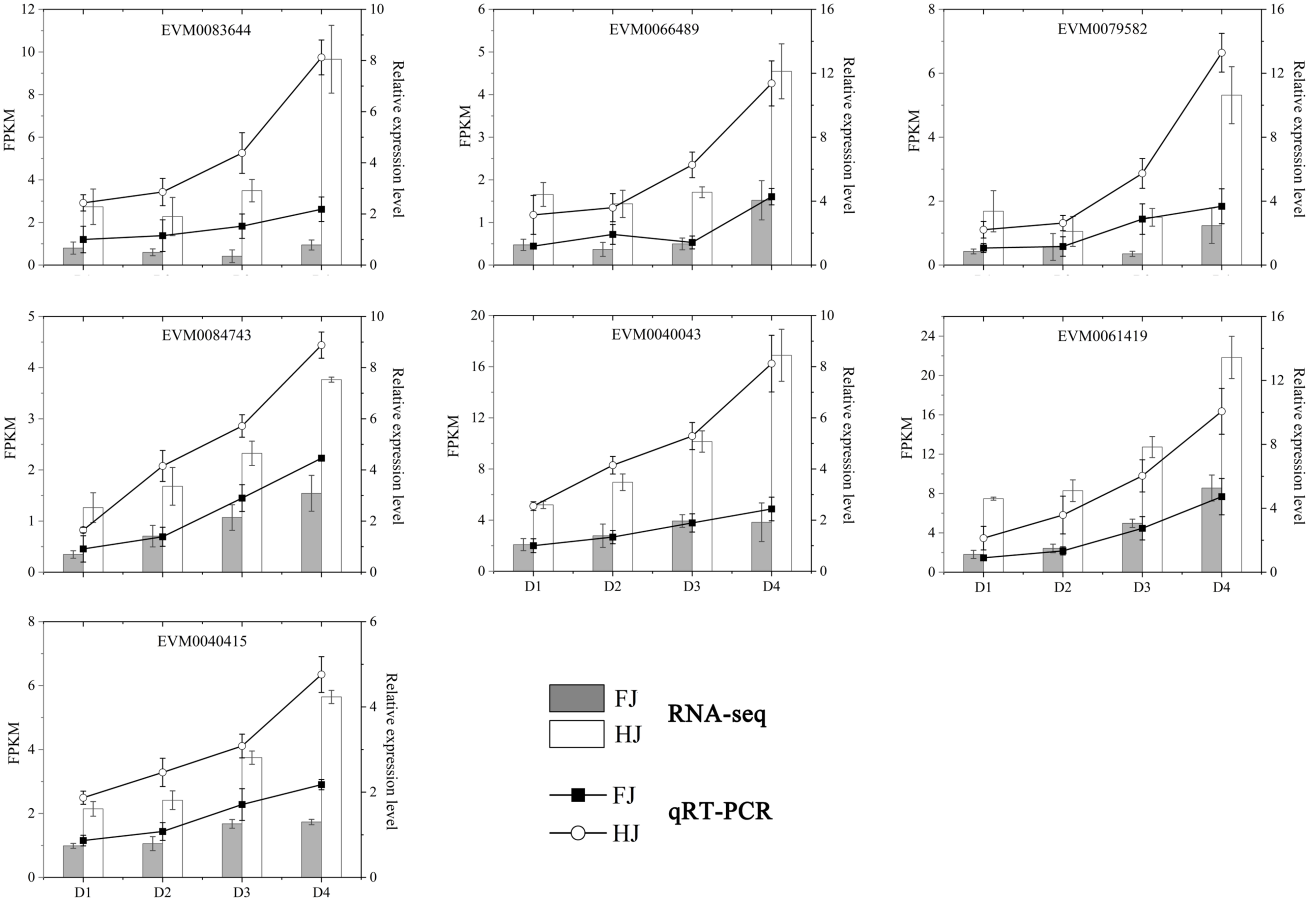
qRT-PCR validation of seven TF genes in co-expression networks. The column chart shows the FPKM values in RNA-seq data. The line diagram represents the relative expression level in qRT-PCR. D1, D2, D3, and D4 indicate 0, 6, 9, and 15 days under drought stress, respectively.

In cyan module, the biosynthesis and metabolism of metabolites were significantly enriched, including flavonoids, terpenoids, amino acids, fatty acids, etc. (Supplementary Figure 4C; Supplementary Table 8). Notably, these metabolites are closely related to enhancing the drought resistance of plants. TFs play important roles in regulating the gene expressions in various life processes. A total of seven TF genes were identified, including 3 *WRKY*s (*WRKY6*, *WRKY33*, *WRKY48*), 2 *BZIP*s (*BZIP23*, *GBF4*), *C2H2* (*IDD1*), and *GRAS* (*SCL14*; Supplementary Table 9). The phylogenetic analysis and *cis*-acting element analysis of these seven TFs were performed (Figure 8D). The phylogenetic analysis showed that the TFs belonging to the same family clustered together, indicating they were similar in sequence structure and molecular function. The *cis*-acting elements were divided into 11 categories, including light responsiveness, drought responsiveness, low-temperature responsiveness and plant hormone responsiveness. The elements enriched in light responsiveness and drought responsiveness were widely existed in the promoters of the seven TFs, implying the important functions of these TFs in *Z. bungeanum* plant photosynthesis under drought stress (Figure 8E). In order to analyze the expression pattern of these seven TF genes, qRT-PCR was performed for two *Z. bungeanum* cultivar leaves under drought conditions. As shown in Figure 9, the relative expression level of these seven TF genes increased steadily and reach the peak value at D4 in both FJ and HJ. Besides, the relative expression level was higher in HJ than in FJ, especially in D3 and D4. The relative expression level in qRT-PCR was consistent to FPKM in RNA-seq, which validated the reliability of our transcriptome data.

## Discussion

Drought negatively affects crop quality and yield, and photosynthesis is an essential metabolic process for carbon assimilation in plants. Therefore, the studies of the effects of drought on photosynthesis have been widely studied in many crops, such as melon (Chevilly et al., 2021), apple (Tan et al., 2017), rice (Todaka et al., 2017), and barley (Daszkowska-Golec et al., 2019). However, in *Z. bungeanum*, the effects of drought on photosynthesis and the underlying molecular mechanism remain unexplored. Here, we monitored photosynthesis indicators in two *Z. bungeanum* cultivars (FJ and HJ) under drought stress and explored the molecular mechanism of photosynthesis changes combining transcriptome data.

Photosynthesis is a complex metabolic process that is affected by multiple factors such as water, CO_2_ concentration, and light (Evans, 2013). The physiological state of plants is also an important limiting factor for photosynthesis. Given that biochemical reactions *in vivo* are catalyzed by enzymes, activities of photosynthesis related enzymes, such as ribulose-1,5-bisphosphate carboxylase/oxygenase, could affect the rate of photosynthesis (Reddy et al., 2004). Besides, photosynthesis is often used as an important evaluation index for the healthy state of plants under abiotic stress conditions. When faced with environmental stress, photosynthesis usually declines sharply and this leads to the inhibition of plant growth and development, which results in a decline in crop quality and yield (Bhagat et al., 2014). In *Z. bungeanum*, the photosynthetic rate increased before 6 d and then decreased dramatically, and a similar pattern was observed for Ci. The initial increase in photosynthesis might stem from the stimulatory effect of abiotic stress. Besides, there was a positive correlation relationship between Pn and Ci (*R* = 0.71, *p* < 0.05). The aforementioned results were in accordance with that the CO_2_ concentration is a key limitation factor to photosynthesis (Wang et al., 2015). Generally speaking, stress-tolerance can protect plants from the disturbance of surrounding environmental stress to a certain extent, which help maintain better quality and higher yield (Sun et al., 2013; Ma et al., 2016; Li et al., 2019). Consistently, we found that drought-tolerant *Z. bungeanum* cultivar HJ exhibited a higher photosynthesis in the late drought period than FJ. Given that sugars play a part in osmotic protection (Sánchez et al., 1998), maintaining higher photosynthesis in turn enhances plant resistance by the provision of more soluble sugars.

Stomata are composed of two guard cells and facilitate gas exchange; they thus play important metabolic roles in processes such as photosynthesis, transpiration, and respiration (Lawson, 2009). Stomatal activity is easily affected by water deficit stress, which could affect the CO_2_ absorption and further influenced the photosynthesis (Osakabe et al., 2014). When suffered water stress, ion- and water-transport systems across membranes regulate the turgor pressure in guard cells and induce stomatal closure (Osakabe et al., 2014). Endogenous ABA produced under drought stress also regulates the stomatal closure by a signal transduction network (Desikan et al., 2004). Morphological observations of stomata displayed that the stomatal aperture of *Z. bungeanum* leaves was significantly decreased after drought stress, which was beneficial to reduce water loss and improve drought tolerance. The promoted stomatal closure was consistent with the research in rice seedlings under drought (Todaka et al., 2017). Notably, the enhanced drought tolerance generally comes at the cost of photosynthesis. The stomatal aperture in HJ was larger than in FJ in the late drought period, which was favorable for maintaining a relatively high photosynthetic efficiency in the HJ. Besides, E was positively correlated with stomatal conductance (*R* = 0.99, *p* < 0.05) and stomatal aperture (*R* = 0.88, *p* < 0.05), supporting that the transpiration rate is directly affected by the stomata (Outlaw and De Vlieghere-He, 2001).

In the photosynthesis process, light energy is captured by LHC proteins and transferred into Calvin-Benson cycle for carbon reduction (Rochaix and Bassi, 2019). In photosynthetic electron transport reactions, the electrons are derived from water and then transferred from PSII to PSI. Thus, the weakened transpiration in *Z. bungeanum* played a negative role in photosynthesis. Chlorophyll *a* and chlorophyll *b* are the major pigments in PSI and PSII, which are responsible for the light energy capture in LHC. Besides, carotenoids play a pivotal role in photoprotection (Bassi and Caffarri, 2000). In this study, the contents of chlorophyll *a*, chlorophyll *b* and carotenoids were decreased in *Z. bungeanum* leaves under drought stress, which caused the decreased photosynthesis rate (Liang et al., 2018; Gao et al., 2020). However, the chlorophyll *a* and chlorophyll *b* contents were higher in HJ than in FJ after 12 days, which may be responsible for the higher photosynthesis in HJ in the late drought stage. Porphyrin and chlorophyll metabolism plays various important physiological roles in plant (Ma et al., 2021). In our study, KEGG analysis revealed that many DEGs were enriched in porphyrin and chlorophyll metabolism, indicated that there was a complex regulation underlying the chlorophyll concentration variation. Additionally, DEGs analysis in F3 vs. H3 and F4 vs. H4 suggested that MAPK signal transduction, the synthesis of brassinosteroid, and the flavonoids metabolism were stronger in HJ than in FJ. As reported, MAPK signal transduction cascades play a crucial role in the response to various abiotic stresses (He et al., 2020). Brassinosteroids widely take part in abiotic stress responses such as drought, salinity, high temperature, low temperature and heavy metal stresses (Li et al., 2021c). As a kind of reductant, flavonoids could contribute to the mitigation of oxidative and enhanced drought tolerance (Nakabayashi et al., 2014; Li et al., 2021a). Taken together, these enhanced pathways may contribute to the higher drought tolerance in HJ.

WGCNA is a powerful tool that has been widely applied into physiological mechanism studies (Yu et al., 2020; Fei et al., 2021). To screen the important genes in photosynthesis, WGCNA was used combining DEGs and physiological indicators in photosynthesis in *Z. bungeanum*. The DEGs in two modules (purple and green) were positively correlated with most photosynthesis indicators. ABA is an important hormone involved in the response to drought stress, and ABA transduction signals play an important role in the activation of TFs and the regulation of the expression of downstream genes (Tuteja, 2007). At the same time, the status of stomata is sensitively regulated by ABA. In the purple module, several genes related to ABA metabolism were identified, including *PYL4* (*EVM0086678*, *EVM0056698*), *PYL9* (*EVM0004250*) and *PYR1* (*EVM0057346*). The dominant ABA-signaling inhibitor (FBPase::abi1-1) promotes biomass accumulation and elevated crop yields in transgenic *Arabidopsis* under drought stress (Negin et al., 2019). Therefore, these genes are thought to be the candidate genes for improving the photosynthesis in *Z. bungeanum* plant. In the co-expression network of the green module, the LHC-encoding gene, *LHCB4.3* (*EVM0014783*), was identified. Given the important role of LHC in light energy capture, the downregulated expression of *LHCB4.3* may decrease light utilization efficiency. In previous studies, inhibition of LCH protein synthesis has been shown to induce photoinhibition in PSII, which in turn caused more ROS accumulation (Pastenes et al., 2005; Guidi et al., 2019). Therefore, *LHCB4.3* was of great significance for the maintenance of photosynthesis and drought resistance in *Z. bungeanum*. In the co-expression network of the purple module, four genes, including *CRD1* (*EVM0071735*), *PORA* (*EVM0025664*) and two *CHLHs* (*EVM0019653*, *EVM0057103*), were selected, and they were enriched in the chlorophyll metabolic pathway, indicating that these four genes may be related to the change of chlorophyll content. However, in-depth research needs to be carried out to prove their function.

TFs are important proteins involved in the regulation of plant growth and development, as well as the response to abiotic stress (Lindemose et al., 2013). Many TFs have been shown to play key roles in the regulation of drought stress and photosynthesis, such as MYB, bZIP and DREB families (Saibo et al., 2009). In cyan module, a total of seven TF genes were identified, including 3 *WRKY*s (*WRKY6*, *WRKY33*, *WRKY48*), 2 *BZIP*s (*BZIP23*, *GBF4*), *C2H2* (*IDD1*) and *GRAS* (*SCL14*) and their expression levels increased under drought stress with higher expression level in HJ than in FJ. *Cis*-acting element analysis of these TFs revealed that a large number of light-responsive and drought-responsive binding sites were distributed in the upstream sequences. In addition, there were also some ABA-responsive binding sites in most TF gene promoters. Together, these results indicated that seven TFs play important roles in the regulation of photosynthesis under drought conditions. Previous research showed that overexpression of the TF encoding gene, *AP37*, elevated the grain yield under drought conditions with enhancing photosynthesis efficiency (Oh et al., 2009). Heterogeneous expression of maize TF *mEmBP-1* enlarged the photosynthesis, biomass, and yield in rice (Perveen et al., 2020). Overexpression of *VuNAC1* and *VuNAC2* promoted growth and stress tolerance by boosting photosynthetic activity in *Arabidopsis* (Srivastava et al., 2022). Therefore, we suspect that these TF genes are of great significance for improving yield of *Z. bungeanum* in the future.

**Figure 10:**
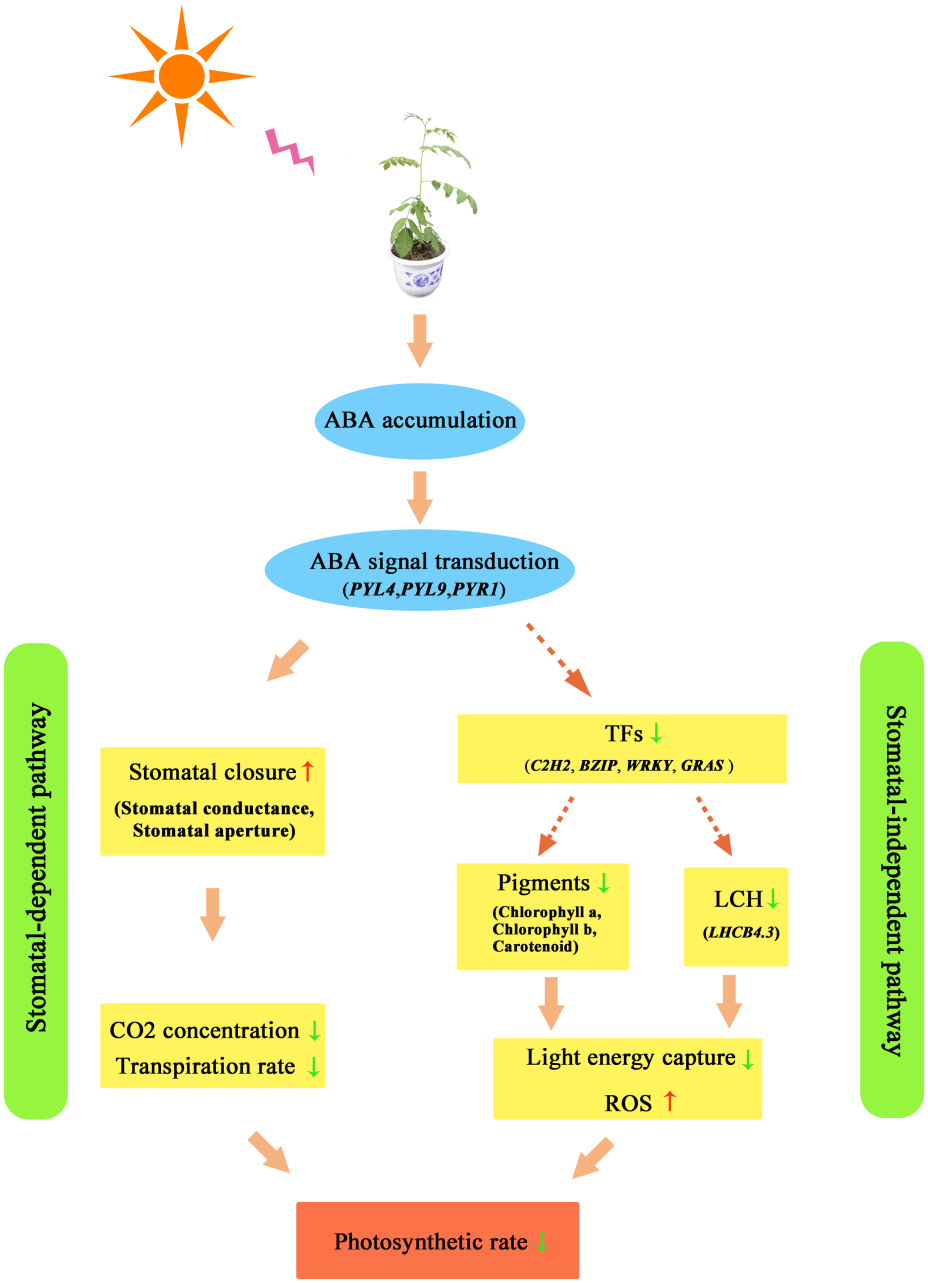
The photosynthesis response model in *Zanthoxylum bungeanum* under drought stress. TF, transcription factor; ROS, reactive oxygen species.

In general, drought caused a decrease in photosynthesis in *Z. bungeanum* leaves through a stomatal-dependent pathway and a stomatal-independent pathway, which is displayed in Figure 10. In the stomatal-dependent pathway, drought-induced ABA accumulation and activated ABA signaling, which resulted in stomatal closure. Then stomatal closure decreased Ci and inhibited E. In a stomatal-independent pathway, drought-induced ABA signaling is transmitted to TF proteins. The TFs affected the light energy capture and transformation efficiency by regulating the expression of genes related to chlorophyll metabolism and LCH protein-encoding genes. The above two pathways jointly led to the decrease in the photosynthetic rate of *Z. bungeanum* under drought conditions.

## Conclusion

In this study, the physiological indicators and molecular mechanisms of photosynthesis under drought stress were explored in two *Z. bungeanum* cultivars with diverse drought-tolerance. Drought resulted in a decrease in photosynthesis due to a decrease in stomatal aperture and Gsw, a descend in Ci and E, and a degradation of chlorophyll and carotenoid. However, in the late drought period, there was a higher photosynthetic rate in HJ, possibly due to higher chlorophyll content, larger stomatal aperture and higher Ci. Through WGCNA analysis, we identified genes involved in ABA signal transduction, LCH-encoding genes, and chlorophyll metabolism genes. In addition, we identified seven TF genes that may be important for regulating photosynthesis under drought conditions. Taken together, our study proposed a model of the photosynthetic in *Z. bungeanum* under drought stress, and the key genes we identified provide important clues for future molecular breeding.

## Data availability statement

The data presented in the study are deposited in the NCBI repository, accession number PRJNA784034.

## Author contributions

HH and AW designed the experiments. HH wrote the manuscript. HH, BH, LM, and XC supervised the research. XF and YLi revised the manuscript. HH, BH, YLu, and PH performed the experiments and data analysis. All authors contributed to the article and approved the submitted version.

## Funding

This study was financially supported by the Technology Innovation Guidance Special Foundation of Shaanxi Province (2020QFY07-01).

## Conflict of interest

The authors declare that the research was conducted in the absence of any commercial or financial relationships that could be construed as a potential conflict of interest.

## Publisher’s note

All claims expressed in this article are solely those of the authors and do not necessarily represent those of their affiliated organizations, or those of the publisher, the editors and the reviewers. Any product that may be evaluated in this article, or claim that may be made by its manufacturer, is not guaranteed or endorsed by the publisher.

## Footnotes

^1^ https://www.biocloud.net

## References

[ref1] Bassi R. Caffarri S. (2000). Lhc proteins and the regulation of photosynthetic light harvesting function by xanthophylls. Photosynth. Res. 64:256. doi: 10.1023/a:1006409506272 16228462

[ref2] Basu S. Ramegowda V. Kumar A. Pereira A. (2016). Plant adaptation to drought stress. F1000Res 5:1554. doi: 10.12688/f1000research.7678.1, PMID: 27441087 PMC4937719

[ref3] Bhagat K. P. Kumar R. A. Ratnakumar P. Kumar S. Bal S. K. Agrawal P. K. (2014). “Photosynthesis and associated aspects Under abiotic stresses environment,” in Approaches to Plant Stress and Their Management. eds. Gaur R. K. Sharma P. (New Delhi: Springer India), 191–205.

[ref4] Chen C. Chen H. Zhang Y. Thomas H. Frank M. He Y.. (2020). TBtools: an integrative toolkit developed for interactive analyses of big biological data. Mol. Plant 13, 1194–1202. doi: 10.1016/j.molp.2020.06.009, PMID: 32585190

[ref5] Chevilly S. Dolz-Edo L. Martínez-Sánchez G. Morcillo L. Vilagrosa A. López-Nicolás J. M.. (2021). Distinctive traits for drought and salt stress tolerance in melon (Cucumis melo L.). front. Plant Sci. 12:777060. doi: 10.3389/fpls.2021.777060, PMID: 34804107 PMC8600367

[ref6] Dai A. (2013). Increasing drought under global warming in observations and models. Nat. Clim. Chang. 3, 52–58. doi: 10.1038/nclimate1633

[ref7] D'Alessandro S. Havaux M. (2019). Sensing β-carotene oxidation in photosystem II to master plant stress tolerance. New Phytol. 223, 1776–1783. doi: 10.1111/nph.15924, PMID: 31090944

[ref8] Daszkowska-Golec A. Collin A. Sitko K. Janiak A. Kalaji H. M. Szarejko I. (2019). Genetic and physiological dissection of photosynthesis in barley exposed to drought stress. Int. J. Mol. Sci. 20:6341. doi: 10.3390/ijms20246341, PMID: 31888211 PMC6940956

[ref9] Desikan R. Cheung M. K. Bright J. Henson D. Hancock J. T. Neill S. J. (2004). ABA, hydrogen peroxide and nitric oxide signalling in stomatal guard cells. J. Exp. Bot. 55, 205–212. doi: 10.1093/jxb/erh033, PMID: 14673026

[ref10] Evans J. R. (2013). Improving photosynthesis. Plant Physiol. 162, 1780–1793. doi: 10.1104/pp.113.219006, PMID: 23812345 PMC3729760

[ref001] Fathi A. Barari D. (2016). Effect of drought stress and its mechanism in plants. Int. J. Life Sci. 10:1. doi: 10.3126/ijls.v10i1.14509

[ref11] Fei X. Qi Y. Lei Y. Wang S. Hu H. Wei A. (2021). Transcriptome and Metabolome dynamics explain aroma differences between green and red prickly ash fruit. Foods 10:391. doi: 10.3390/foods10020391, PMID: 33579038 PMC7916813

[ref12] Fei X. Shi Q. Yang T. Fei Z. Wei A. (2018). Expression stabilities of ten candidate reference genes for RT-qPCR in *Zanthoxylum bungeanum* maxim. Molecules 23:802. doi: 10.3390/molecules23040802, PMID: 29601541 PMC6017173

[ref13] Feng S. Liu Z. Hu Y. Tian J. Yang T. Wei A. (2020). Genomic analysis reveals the genetic diversity, population structure, evolutionary history and relationships of Chinese pepper. Hortic. Res. 7:158. doi: 10.1038/s41438-020-00376-z, PMID: 33082965 PMC7527552

[ref14] Gao T. Zhang Z. Liu X. Wu Q. Chen Q. Liu Q.. (2020). Physiological and transcriptome analyses of the effects of exogenous dopamine on drought tolerance in apple. Plant Physiol. Biochem. 148, 260–272. doi: 10.1016/j.plaphy.2020.01.022, PMID: 31982861

[ref15] Guidi L. Lo Piccolo E. Landi M. (2019). Chlorophyll fluorescence, Photoinhibition and abiotic stress: does it make Any difference the fact to be a C3 or C4 species? Front. Plant Sci. 10:174. doi: 10.3389/fpls.2019.00174, PMID: 30838014 PMC6382737

[ref16] Gururani M. A. Venkatesh J. Tran L. S. P. (2015). Regulation of photosynthesis during abiotic stress-induced Photoinhibition. Mol. Plant 8, 1304–1320. doi: 10.1016/j.molp.2015.05.005, PMID: 25997389

[ref17] He X. Wang C. Wang H. Li L. Wang C. (2020). The function of MAPK cascades in response to various stresses in horticultural plants. Front. Plant Sci. 11:952. doi: 10.3389/fpls.2020.00952, PMID: 32849671 PMC7412866

[ref18] Hong Y. Wang Z. Liu X. Yao J. Kong X. Shi H.. (2020). Two chloroplast proteins negatively regulate plant drought resistance Through separate pathways. Plant Physiol. 182, 1007–1021. doi: 10.1104/pp.19.01106, PMID: 31776182 PMC6997674

[ref19] Huang Z. Shen L. Wang W. Mao Z. Yi X. Kuang T.. (2021). Structure of photosystem I-LHCI-LHCII from the green alga Chlamydomonas reinhardtii in state 2. Nat. Commun. 12:1100. doi: 10.1038/s41467-021-21362-6, PMID: 33597543 PMC7889890

[ref20] Kaiser E. Morales A. Harbinson J. Kromdijk J. Heuvelink E. Marcelis L. F. (2015). Dynamic photosynthesis in different environmental conditions. J. Exp. Bot. 66, 2415–2426. doi: 10.1093/jxb/eru406 25324402

[ref21] Kim D. Langmead B. Salzberg S. L. (2015). HISAT: a fast spliced aligner with low memory requirements. Nat. Methods 12, 357–360. doi: 10.1038/nmeth.3317, PMID: 25751142 PMC4655817

[ref22] Langfelder P. Horvath S. (2008). WGCNA: an R package for weighted correlation network analysis. BMC Bioinformatics 9:559. doi: 10.1186/1471-2105-9-559, PMID: 19114008 PMC2631488

[ref23] Lawson T. (2009). Guard cell photosynthesis and stomatal function. New Phytol. 181, 13–34. doi: 10.1111/j.1469-8137.2008.02685.x 19076715

[ref24] Li B. Fan R. Sun G. Sun T. Fan Y. Bai S.. (2021a). Flavonoids improve drought tolerance of maize seedlings by regulating the homeostasis of reactive oxygen species. Plant and Soil 461, 389–405. doi: 10.1007/s11104-020-04814-8

[ref25] Li P. Ruan Z. Fei Z. Yan J. Tang G. (2021b). Integrated Transcriptome and Metabolome analysis revealed That flavonoid biosynthesis may dominate the resistance of *Zanthoxylum bungeanum* against stem canker. J. Agric. Food Chem. 69, 6360–6378. doi: 10.1021/acs.jafc.1c00357, PMID: 34043342

[ref26] Li S. Y. Wang W. B. Yao X. D. Wang C. L. Cao Y. Q. Zhang L. J.. (2019). Photosynthesis in reciprocal grafts of drought-tolerant and drought-sensitive cultivars of soybean under water stress. Photosynthetica 57, 942–949. doi: 10.32615/ps.2019.109

[ref27] Li S. Zheng H. Lin L. Wang F. Sui N. (2021c). Roles of brassinosteroids in plant growth and abiotic stress response. Plant Growth Regul. 93, 29–38. doi: 10.1007/s10725-020-00672-7

[ref28] Liang B. Ma C. Zhang Z. Wei Z. Gao T. Zhao Q.. (2018). Long-term exogenous application of melatonin improves nutrient uptake fluxes in apple plants under moderate drought stress. Environ. Exp. Bot. 155, 650–661. doi: 10.1016/j.envexpbot.2018.08.016

[ref29] Lima-Melo Y. Kılıç M. Aro E. M. Gollan P. J. (2021). Photosystem I inhibition, protection and Signalling: Knowns and unknowns. Front. Plant Sci. 12:791124. doi: 10.3389/fpls.2021.791124, PMID: 34925429 PMC8671627

[ref30] Lindemose S. O'Shea C. Jensen M. K. Skriver K. (2013). Structure, function and networks of transcription factors involved in abiotic stress responses. Int. J. Mol. Sci. 14, 5842–5878. doi: 10.3390/ijms14035842, PMID: 23485989 PMC3634440

[ref31] Ma X. Xia H. Liu Y. Wei H. Zheng X. Song C.. (2016). Transcriptomic and Metabolomic studies disclose key metabolism pathways contributing to well-maintained photosynthesis under the drought and the consequent drought-tolerance in Rice. Front. Plant Sci. 7:1886. doi: 10.3389/fpls.2016.01886, PMID: 28066455 PMC5174129

[ref32] Ma Q. Xu X. Xie Y. Huang T. Wang W. Zhao L.. (2021). Comparative metabolomic analysis of the metabolism pathways under drought stress in alfalfa leaves. Environ. Exp. Bot. 183:104329. doi: 10.1016/j.envexpbot.2020.104329

[ref33] Nakabayashi R. Mori T. Saito K. (2014). Alternation of flavonoid accumulation under drought stress in Arabidopsis thaliana. Plant Signal. Behav. 9:e29518. doi: 10.4161/psb.29518, PMID: 25763629 PMC4203635

[ref34] Negin B. Yaaran A. Kelly G. Zait Y. Moshelion M. (2019). Mesophyll Abscisic acid restrains early growth and flowering But does not directly suppress photosynthesis. Plant Physiol. 180, 910–925. doi: 10.1104/pp.18.01334, PMID: 30910907 PMC6548251

[ref35] Oh S.-J. Kim Y. S. Kwon C.-W. Park H. K. Jeong J. S. Kim J.-K. (2009). Overexpression of the transcription factor AP37 in Rice improves grain yield under drought conditions. Plant Physiol. 150, 1368–1379. doi: 10.1104/pp.109.137554, PMID: 19429605 PMC2705040

[ref36] Okagu I. U. Ndefo J. C. Aham E. C. Udenigwe C. C. (2021a). Zanthoxylum species: A comprehensive review of traditional uses, Phytochemistry, pharmacological and Nutraceutical applications. Molecules 26:4023. doi: 10.3390/molecules26134023, PMID: 34209371 PMC8272177

[ref37] Okagu I. U. Ndefo J. C. Aham E. C. Udenigwe C. C. (2021b). Zanthoxylum species: A review of traditional uses, Phytochemistry and pharmacology in relation to cancer, infectious diseases and sickle cell anemia. Front. Pharmacol. 12:713090. doi: 10.3389/fphar.2021.713090, PMID: 34603027 PMC8479109

[ref38] Osakabe Y. Osakabe K. Shinozaki K. Tran L.-S. P. (2014). Response of plants to water stress. Front. Plant Sci. 5:86. doi: 10.3389/fpls.2014.00086, PMID: 24659993 PMC3952189

[ref39] Outlaw W. H. De Vlieghere-He X. (2001). Transpiration rate. An important factor controlling the sucrose content of the guard cell apoplast of broad bean. Plant Physiol. 126, 1716–1724. doi: 10.1104/pp.126.4.1716, PMID: 11500569 PMC117170

[ref40] Pastenes C. Pimentel P. Lillo J. (2005). Leaf movements and photoinhibition in relation to water stress in field-grown beans. J. Exp. Bot. 56, 425–433. doi: 10.1093/jxb/eri061, PMID: 15596474

[ref41] Pertea M. Pertea G. M. Antonescu C. M. Chang T.-C. Mendell J. T. Salzberg S. L. (2015). String tie enables improved reconstruction of a transcriptome from RNA-seq reads. Nat. Biotechnol. 33, 290–295. doi: 10.1038/nbt.3122, PMID: 25690850 PMC4643835

[ref42] Perveen S. Qu M. Chen F. Essemine J. Khan N. Lyu M.-J. A.. (2020). Overexpression of maize transcription factor mEmBP-1 increases photosynthesis, biomass, and yield in rice. J. Exp. Bot. 71, 4944–4957. doi: 10.1093/jxb/eraa248, PMID: 32442255

[ref43] Reddy A. R. Chaitanya K. V. Vivekanandan M. (2004). Drought-induced responses of photosynthesis and antioxidant metabolism in higher plants. J. Plant Physiol. 161, 1189–1202. doi: 10.1016/j.jplph.2004.01.013 15602811

[ref44] Rochaix J.-D. (2013). Fine-tuning photosynthesis. Science 342, 50–51. doi: 10.1126/science.1244943, PMID: 24092720 PMC4317468

[ref45] Rochaix J.-D. Bassi R. (2019). LHC-like proteins involved in stress responses and biogenesis/repair of the photosynthetic apparatus. Biochem. J. 476, 581–593. doi: 10.1042/BCJ20180718, PMID: 30765616

[ref46] Saibo N. J. M. Lourenço T. Oliveira M. M. (2009). Transcription factors and regulation of photosynthetic and related metabolism under environmental stresses. Ann. Bot. 103, 609–623. doi: 10.1093/aob/mcn227, PMID: 19010801 PMC2707349

[ref47] Sánchez F. J. Manzanares M. A. de Andres E. F. Tenorio J. L. Ayerbe L. (1998). Turgor maintenance, osmotic adjustment and soluble sugar and proline accumulation in 49 pea cultivars in response to water stress. Field Crop Res 59, 225–235. doi: 10.1016/S0378-4290(98)00125-7

[ref48] Schmittgen T. D. Livak K. J. (2008). Analyzing real-time PCR data by the comparative CT method. Nat. Protoc. 3, 1101–1108. doi: 10.1038/nprot.2008.73, PMID: 18546601

[ref49] Srivastava R. Kobayashi Y. Koyama H. Sahoo L. (2022). Overexpression of cowpea NAC transcription factors promoted growth and stress tolerance by boosting photosynthetic activity in Arabidopsis. Plant Sci. 319:111251. doi: 10.1016/j.plantsci.2022.111251, PMID: 35487661

[ref50] Sun J. Gu J. Zeng J. Han S. Song A. Chen F.. (2013). Changes in leaf morphology, antioxidant activity and photosynthesis capacity in two different drought-tolerant cultivars of chrysanthemum during and after water stress. Sci. Hortic. 161, 249–258. doi: 10.1016/j.scienta.2013.07.015

[ref51] Sun M. Peng F. Xiao Y. Yu W. Zhang Y. Gao H. (2020). Exogenous phosphatidylcholine treatment alleviates drought stress and maintains the integrity of root cell membranes in peach. Sci. Hortic. 259:108821. doi: 10.1016/j.scienta.2019.108821

[ref52] Tan Y. Li M. Yang Y. Sun X. Wang N. Liang B.. (2017). Overexpression of MpCYS4, A Phytocystatin Gene from Malus prunifolia (Willd.) Borkh., enhances Stomatal closure to confer drought tolerance in transgenic Arabidopsis and apple. Front. Plant Sci. 8:33. doi: 10.3389/fpls.2017.00033, PMID: 28174579 PMC5258747

[ref53] Tian J. Ma Y. Tian L. Huang C. Chen M. Wei A. (2021). Comparative physiology and transcriptome response patterns in cold-tolerant and cold-sensitive varieties of *Zanthoxylum bungeanum* maxim. Ind. Crop Prod. 167:113562. doi: 10.1016/j.indcrop.2021.113562

[ref54] Todaka D. Zhao Y. Yoshida T. Kudo M. Kidokoro S. Mizoi J.. (2017). Temporal and spatial changes in gene expression, metabolite accumulation and phytohormone content in rice seedlings grown under drought stress conditions. Plant J. 90, 61–78. doi: 10.1111/tpj.13468, PMID: 28019048

[ref55] Tuteja N. (2007). Abscisic acid and abiotic stress signaling. Plant Signal. Behav. 2, 135–138. doi: 10.4161/psb.2.3.4156, PMID: 19516981 PMC2634038

[ref56] van Bezouwen L. S. Caffarri S. Kale R. S. Kouřil R. Thunnissen A.-M. W. H. Oostergetel G. T.. (2017). Subunit and chlorophyll organization of the plant photosystem II supercomplex. Nat. Plants 3:17080. doi: 10.1038/nplants.2017.80, PMID: 28604725

[ref57] Wang C. Han F. Chen X. Zhao A. Wang D. (2022). Time-series based metabolomics reveals the characteristics of the color-related metabolites during the different coloration stages of *Zanthoxylum bungeanum* peel. Food Res. Int. 155:111077. doi: 10.1016/j.foodres.2022.111077, PMID: 35400454

[ref58] Wang Y. Stessman D. J. Spalding M. H. (2015). The CO2 concentrating mechanism and photosynthetic carbon assimilation in limiting CO2: how Chlamydomonas works against the gradient. Plant J. 82, 429–448. doi: 10.1111/tpj.12829, PMID: 25765072

[ref59] Xue Q. Fan H. Yao F. Cao X. Liu M. Sun J.. (2020). Transcriptomics and targeted metabolomics profilings for elucidation of pigmentation in Lonicera japonica flowers at different developmental stages. Ind. Crop Prod. 145:111981. doi: 10.1016/j.indcrop.2019.111981

[ref60] Yu B. Liu J. Wu D. Liu Y. Cen W. Wang S.. (2020). Weighted gene coexpression network analysis-based identification of key modules and hub genes associated with drought sensitivity in rice. BMC Plant Biol. 20:478. doi: 10.1186/s12870-020-02705-9, PMID: 33081724 PMC7576772

[ref61] Zhang A. Liu M. Gu W. Chen Z. Gu Y. Pei L.. (2021). Effect of drought on photosynthesis, total antioxidant capacity, bioactive component accumulation, and the transcriptome of Atractylodes lancea. BMC Plant Biol. 21:293. doi: 10.1186/s12870-021-03048-9, PMID: 34171994 PMC8226357

[ref62] Zhang M. Wang J. Zhu L. Li T. Jiang W. Zhou J.. (2017). *Zanthoxylum bungeanum* maxim. (Rutaceae): A systematic review of its traditional uses, botany, Phytochemistry, pharmacology, pharmacokinetics, and toxicology. Int. J. Mol. Sci. 18:2172. doi: 10.3390/ijms18102172, PMID: 29057808 PMC5666853

[ref63] Zhang H. Zhu J. Gong Z. Zhu J. K. (2022). Abiotic stress responses in plants. Nat. Rev. Genet. 23, 104–119. doi: 10.1038/s41576-021-00413-0 34561623

