# Supplementary material for: Physiological and transcriptome analyses reveal the photosynthetic response to drought stress in drought-sensitive (Fengjiao) and drought-tolerant (Hanjiao) Zanthoxylum bungeanum cultivars
Source: Front Plant Sci. 2022 Sep 6;13:968714. doi: 10.3389/fpls.2022.968714 (PMC9524374; doi:10.3389/fpls.2022.968714)
Supplement: Supplementary file 2 [file Data_Sheet_2.pdf]

Supplementary Material

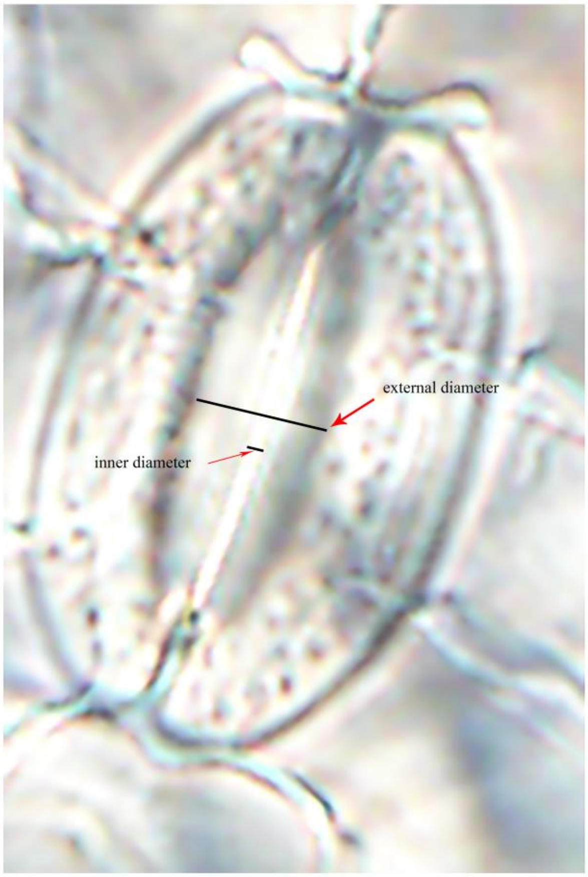

Note: Inner diameter was defined as stomatal aperture.

Supplementary Figure 1. The measurement of stomatal aperture. The inner diameter was defined as stomatal aperture in leaves.

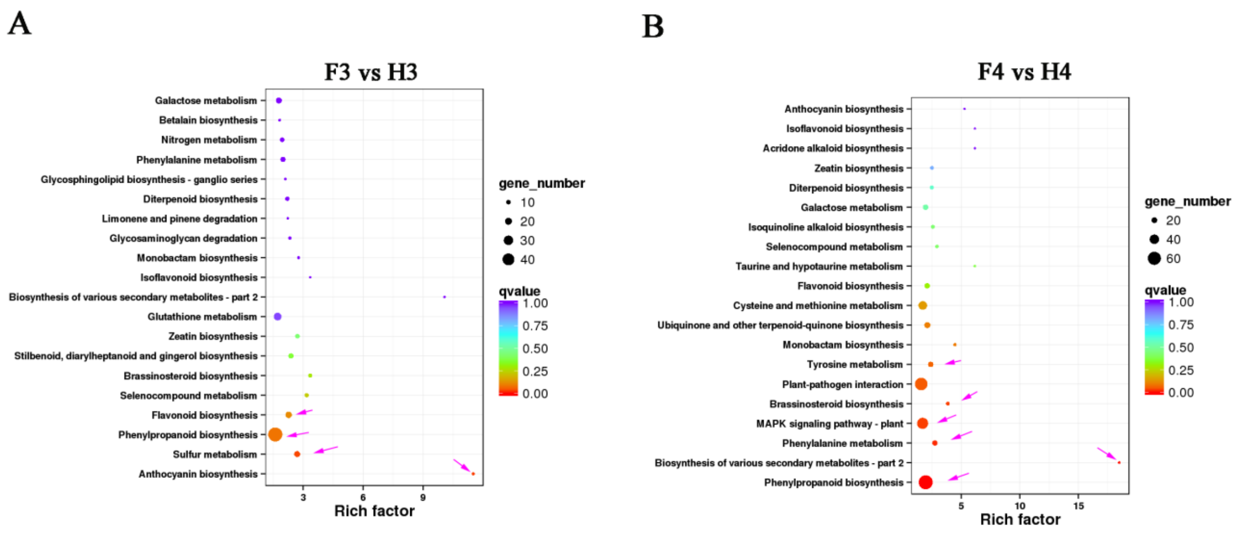

Supplementary Figure 2. The KEGG enrichment analysis of up-regulated genes in F3 vs. H3 (A) and F4 vs. H4 (B). Pink arrows pointed to significantly enriched pathways, which were associated with drought response. F3, and F4 indicate FJ samples in 9d and 15d under drought stress; H3 and H4 indicate HJ samples in 9 and 15d under drought stress.

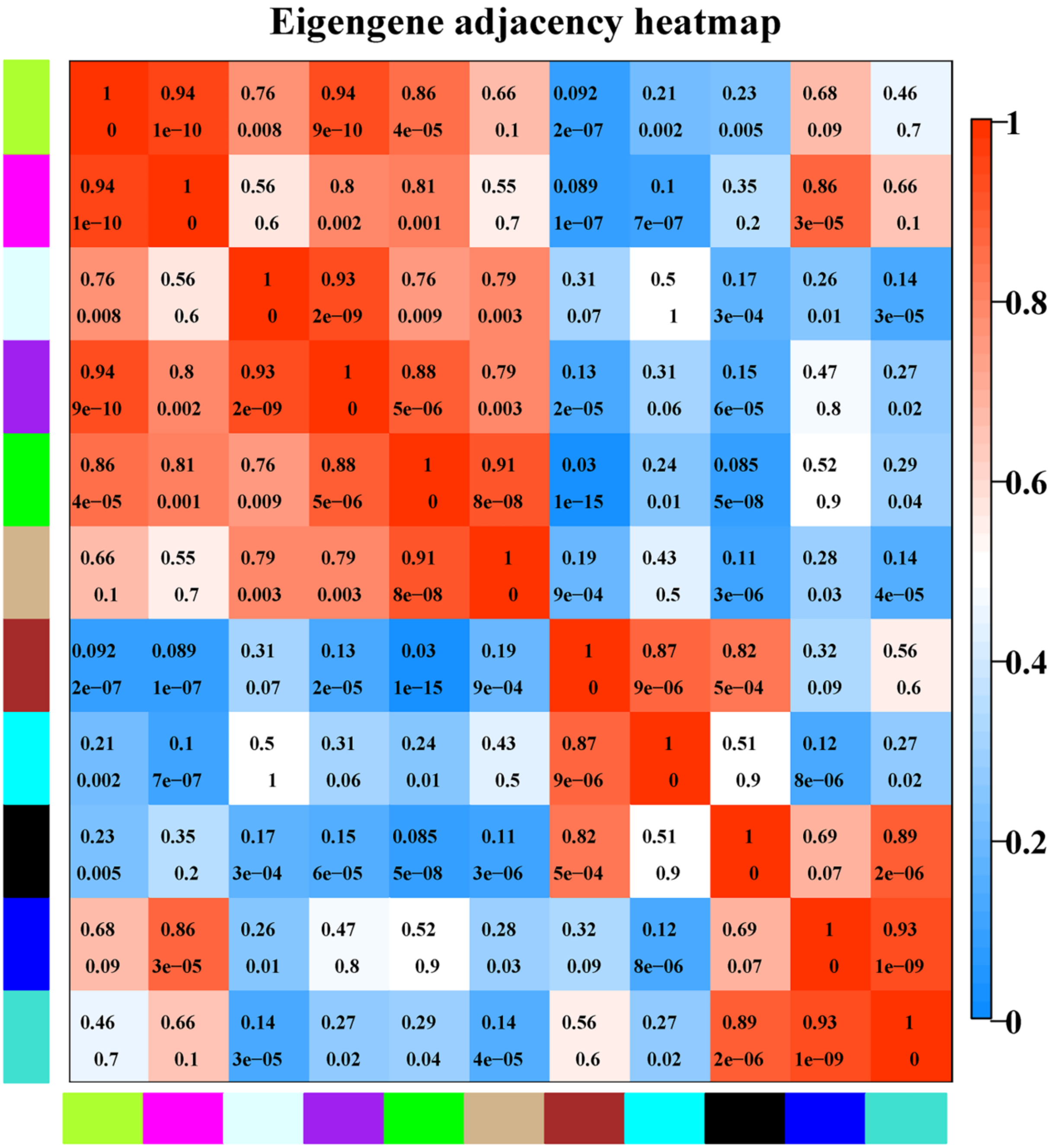

Supplementary Figure 3. Adjacency heatmap of eleven modules in weighted gene co-expression network analysis.

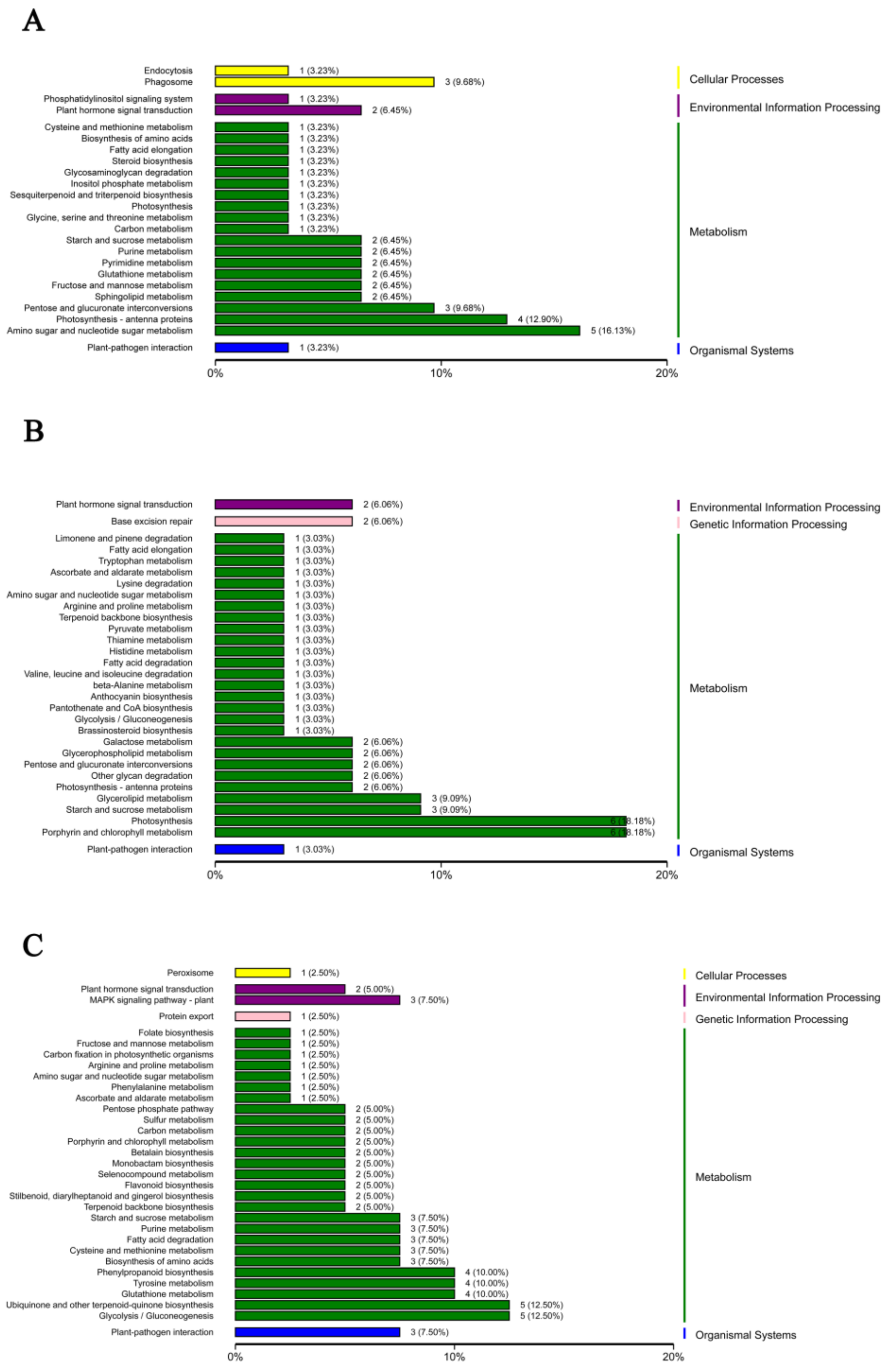

Supplementary Figure 4. KEGG annotations of 150 core genes in the green (A), purple (B) and cyan modules (C).

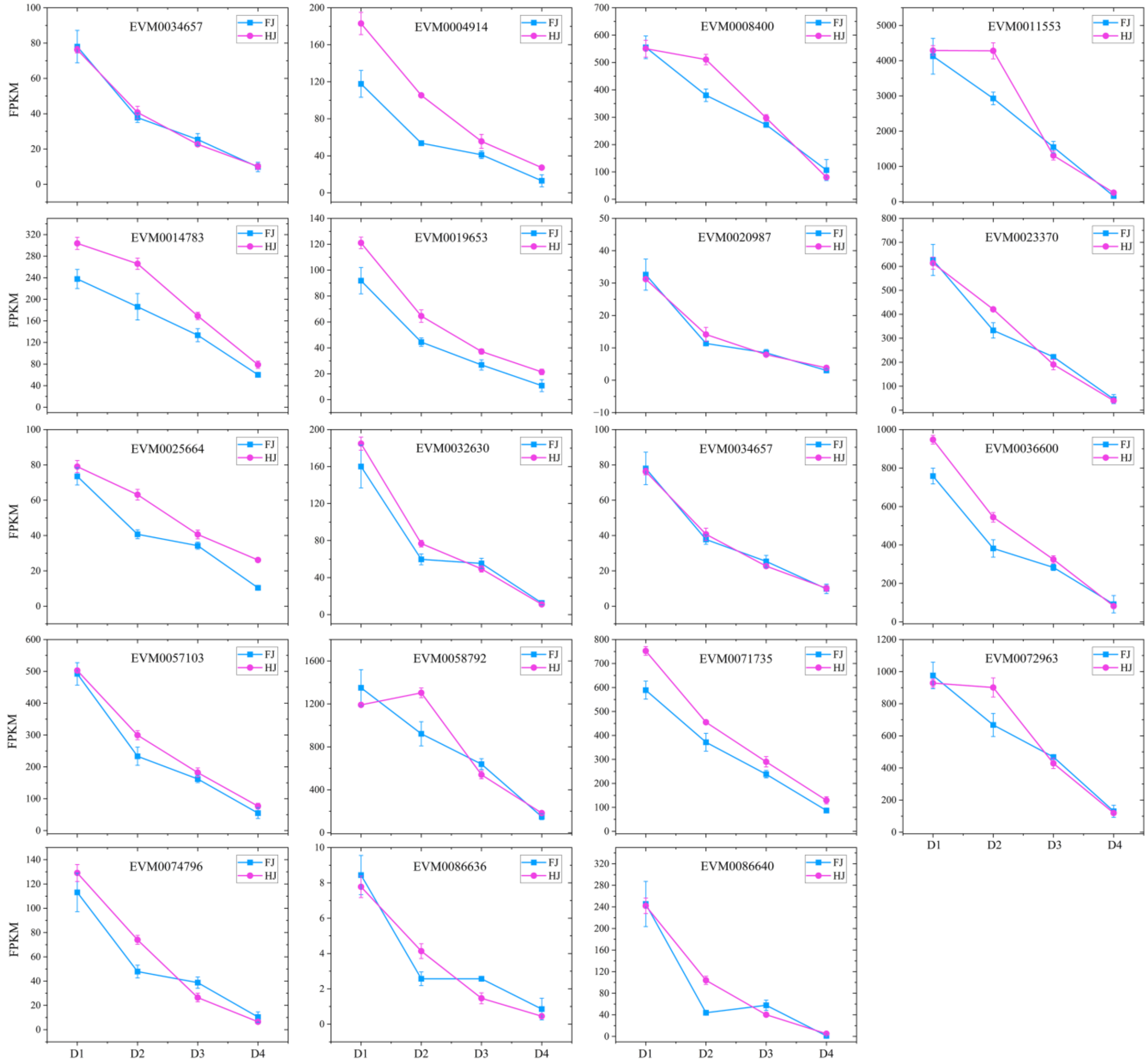

Supplementary Figure 5. The expression level of 19 selected photosynthesis genes in green and purple co-expression networks. D1, D2, D3, and D4 indicate 0d, 6d, 9d and 15d under drought stress, respectively.
